# Nanoscale ZnO/α‐Fe_2_O_3_ Heterostructures: Toward Efficient and Low‐Cost Photoanodes for Water Splitting

**DOI:** 10.1002/smsc.202100104

**Published:** 2021-12-01

**Authors:** Letizia Liccardo, Edlind Lushaj, Laura Dal Compare, Elisa Moretti, Alberto Vomiero

**Affiliations:** ^1^ Department of Molecular Sciences and Nanosystems Ca’ Foscari University of Venice Via Torino 155 30172 Venezia Mestre Italy; ^2^ Division of Materials Science Department of Engineering Sciences and Mathematics Luleå University of Technology 97187 Luleå Sweden

**Keywords:** composite ZnO/α-Fe_2_O_3_, hierarchical nanostructures, nanostructured ZnO, nanostructured α-Fe_2_O_3_, photoanodes, photoelectrochemical water splitting

## Abstract

Composite metal oxide semiconductors are promising candidates for photoelectrochemical water splitting (PEC WS) toward environmentally friendly hydrogen production. Among them, ZnO and α‐Fe_2_O_3_ hold great potential thanks to a series of benefits, including fast charge transport in single‐crystalline structures, large surface area and tunable shapes (ZnO), and energy bandgap falling in the visible spectral range (α‐Fe_2_O_3_). However, both materials present significant drawbacks, which hinder their successful application in high‐efficiency PEC WS: the wide bandgap of ZnO limits its absorption in the UV range, while the low charge carrier mobility results in heavy recombination losses in α‐Fe_2_O_3_ during charge collection. The synthesis of ZnO/hematite composites has recently proven to be an effective approach to improve the overall WS performances. In this review, the recent developments on the application of different morphologies (0D, 1D, 2D, and 3D structures) for PEC WS are illustrated, analyzing the role of the shape and morphology in boosting the functional properties, both in single systems and in composite nanostructures. Complex networks show higher photocatalytic efficiency than the single building blocks and, consequently, composite materials exhibit higher performances. Possible paths for the development of an effective lab‐to‐fab transition based on application of ZnO/α‐Fe_2_O_3_ composite structures are also suggested.

## Graphical abstract

ZnO/hematite composite nanostructures are promising candidates for photoelectrochemical water splitting. Recent advancements demonstrate that the combination of single building blocks and the designing of complex morphologies may enhance the performances of both single and composite systems. The shape of a material can be a key parameter in producing new catalysts for energy and environmental applications.

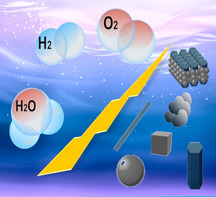

## 1 Introduction

In 2018, world total electricity final consumption reached 22 315 TWh. During the same year, world gross electricity production increased by 3.9%, and the 66.3% of total production was given by fuels, which includes coal and coal products, oil and oil products, natural gas, biofuels including biomasses, industrial waste, and municipal waste.^[^ ^1^ ^]^

Global energy consumption increased by an average of 2% annually until 2018, compared to the slowdown in growth in 2019–2020, in a context of economic crisis due to the COVID‐19 pandemic situation. In fact, global energy demand fell 3.8% in early 2020, with most of the effects felt in March, when confinement measures were imposed in Europe, North America, and elsewhere. Consequently, a drop in CO_2_ (8.8%) emissions was observed in the first half of 2020.^[^ ^2^ ^]^ It is an historical decline, but a possible rebound effect of the CO_2_ emissions in 2021 can occur, depending on economic recovery and people mobility.

Therefore, our society is facing the need of energy and its production in a sustainable way.^[^ ^3^ ^]^ It is essential to shift energy systems away from fossil fuels toward renewable sources, not only to satisfy the increasing energy demand, due to the rapid population growth, but also to reduce drastically the CO_2_ global emissions, which cause severe climate changes.

Among all, hydrogen is a promising clean fuel due to its high energy density/mass ratio (120–142 MJ kg^−1^) with zero CO_2_ emissions. One of the methods allowing H_2_ production is the splitting of water into hydrogen and oxygen, according to Equation (1).^[^ 4, 5, 6, 7, 8 ^]^

(1) $$2H_{2}O\to O_{2}+2H_{2}$$

**Figure 1:**
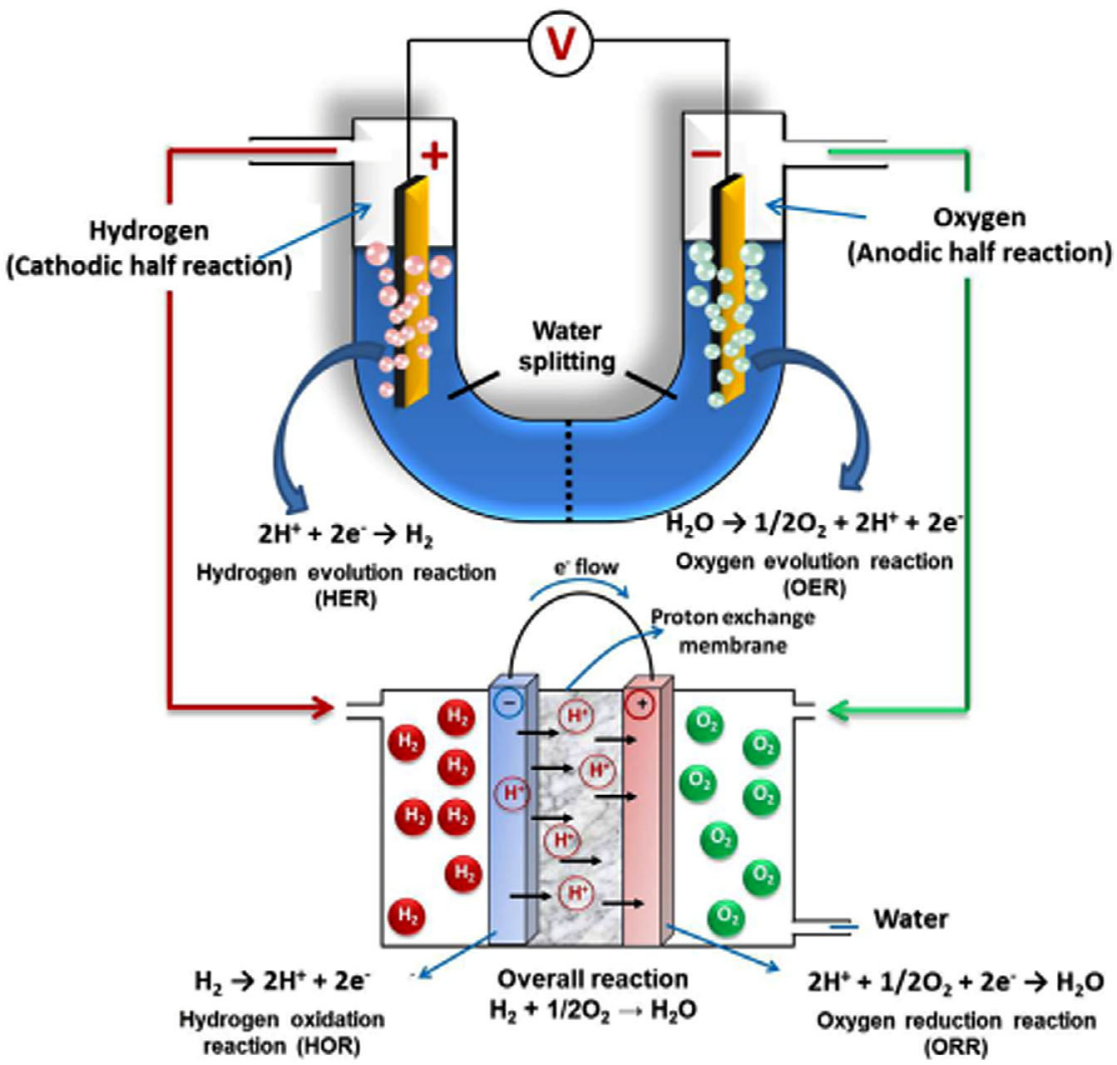
Schematic representation of oxygen reduction reaction (ORR) in fuel cell, OER, HER, and hydrogen oxidation reaction (HOR) by water hydrolysis. Reproduced with permission.^[^ ^13^ ^]^ Copyright 2017, American Chemical Society.

Electrochemical water splitting (WS) has been regarded as a clean and promising approach for the production of hydrogen under mild conditions.^[^ 9, 10 ^]^ Two half reactions, hydrogen evolution reaction (HER, Equation (2)) at the cathode and oxygen evolution reaction (OER, Equation (3)) at the anode, are involved in the process, which occurs in an electrolysis cell made up of anode, cathode, and an aqueous electrolyte (**Figure** **1**).^[^ 4, 5, 6, 7, 8, 9, 10, 11, 12, 13 ^]^

(2) $$\text{Acidic HER}:4e^{-}+4H^{+}\to 2H_{2}$$

(3) $$\text{Basic HER}:4H_{2}O+4e^{-}\to 2H_{2}+4{\text{OH}}^{-}$$

(4) $$\text{Acidic OER}:2H_{2}O\to O_{2}+4e^{-}+4H^{+}$$

(5) $$\text{Basic OER}:4{\text{OH}}^{-}\to O_{2}+2H_{2}O+4e^{-}$$

To carry out the WS process, a potential higher than the theoretical thermodynamic potential (1.23 V at 25 °C and 1 atm) is required.^[^ 6, 12 ^]^ This excess potential, called overpotential, is usually quite large because commercial electrolyzers typically operate at a cell voltage of 1.8–2.0 V, thus limiting its practical use.^[^ 13, 14 ^]^ Therefore, decreasing the overpotential is key to make this process highly efficient.^[^ ^15^ ^]^ First of all, highly active hydrogen evolution and oxygen evolution catalysts should be used to produce large current density. Furthermore, they should display long‐term stability.^[^ 11, 13 ^]^ Currently, Pt‐group metals, and Ir‐ and Ru‐based compounds have the highest HER and OER activity, respectively. However, they may be too costly and scarce for widespread applications.^[^ 12, 13, 14, 15, 16, 17 ^]^

Solar energy is the most abundant of all the nonrenewable and renewable sources and the energy supplied by the sun to the earth (173.000 TW) is about 9.600 times larger than the daily total global energy consumption. For this reason, exploiting solar energy for photoelectrochemical (PEC) WS would be extremely important. In 1972, Fujishima and Honda^[^ ^18^ ^]^ demonstrated that the use of such chemical reaction is one of the most promising approaches to convert solar energy into clean hydrogen fuels.^[^ 19, 20, 21 ^]^

From a thermodynamic point of view, PEC is a penalized reaction needing energy to overcome a potential barrier given by a Gibbs free energy variation of 237 kJ mol^−1^.^[^ 22, 23, 24 ^]^

For electrochemical WS, the PEC cell is usually composed of a photoanode and a cathode in an aqueous environment, within which an electrolyte is found.^[^ ^25^ ^]^ Moreover, semiconductor materials are needed at electrodes to convert photons into chemical energy.^[^ 26, 27 ^]^ The energy needed to overcome the potential barrier is provided to the semiconductor through photons with energies greater or equal to the energy bandgap (*E*_g_). This process causes the electrons to be excited toward the conduction band (CB) and, consequently, to generate holes in the valence band (VB).^[^ ^20^ ^]^ It is required for the VB potential to be more positive than the O_2_/H_2_O redox potential to allow for water oxidation, while for the CB to be more negative than H^+^/H_2_ redox potential to allow for water reduction.^[^ 20, 23 ^]^

Nowadays, many efforts are in place to design highly efficient, stable, and low‐cost photoelectrodes with tailored and tunable electronic properties. Highly efficient solar‐chemical energy conversion is possible if the design of novel photoelectrodes overcomes some critical points: 1) photogenerated electron (e^−^) and hole (h^+^) pairs must reach the catalyst surface rapidly and the diffusion lengths should be as long as possible to avoid their fast recombination; 2) photoelectrode specific surface area should increase to enhance the adsorption of water molecules; and 3) surface reactions kinetics should be high.^[^ ^28^ ^]^ Therefore, the best semiconductor for PEC WS must show specific properties. First, its bandgap should be lower than 3.0 eV to have a wide range of solar light absorption, but larger than 1.23 eV to have proper band‐edge potentials for water‐splitting reaction. The CB potential should be more negative than the thermodynamic potential for water reduction, while the VB potential more positive than that for water oxidation.^[^ ^29^ ^]^ Finally, the appropriate semiconductor should have excellent stability in photochemical reactions.^[^ ^30^ ^]^ Researchers in the field of EC and PEC WS are now focusing on the use of sulfides,^[^ 31, 32 ^]^ metal oxides,^[^ 33, 34, 35 ^]^ noble metal,^[^ ^36^ ^]^ and nonmetal^[^ 37, 38 ^]^ semiconductors (**Table** **1**).

**Table 1:** Efficient photoelectrodes reported for WS. Readapted and updated from ref. [39]

Photoactive material		Cocatalyst	Electrolyte	Stability [h]	*J*_sc_ [mA cm^−2^] at 0 V versus RHE	STH efficiency	Ref.
A. Highly efficient PEs (STH > 10%)							
1	p‐GaInP_2_/GaAs	Pt	3 m H_2_SO_4_	3	120	12.4	[40]
2	AlGaAs/Si	RuO_2_/Pt	1 m H_2_SO_4_	–	20.1	18.3	[41]
3	Sb_2_Se_3_/CdS/TiO_2_	Pt	Near‐neutral buffered solution	10	8.6	10.6	[42]
B. Acceptable PEs (STH 4–10%)							
4	Cu_2_O/Al_2_O_3_/TiO_2_	Pt/MoS_2_	0.1 m NaClO_4_	5	5.7	7	[43]
5	Si pillars	Mo_3_S_4_	1 m HClO_4_	1	9	10	[44]
6	p‐Si	Fe_2_O_3_/Au NPs	1 m NaOH	0.5	2.6	6	[45]
7	CdS/Cu(In,GaS_2_)	Pt/Mo/Ti	Complex	7	30	8.5	[46]
8	Au/TiO_2_	NiCr	1 m NaOH	1	9	4.0	[47]
9	AgSbS_2_‐ZnO	—	1 m Na_2_S	2	5.8	5.76	[48]
10	CuSbS_2_/CdS	Pt	Near‐neutral buffered solution	1	5.2	4.2	[49]
C. Marginal PEs (STH > 1%)							
11	p‐Si/SiO_2_	Ti	3 m H_2_SO_4_	2	20	2.9	[50]
12	CdS/CuGaSe_2_	Pt/Ag	1 m Na_2_SO_4_	8	8.1	1.2	[51]
13	CdS/CuGa_3_Se_3_	Pt/Ag	0.1 m Na_2_HPO_4_	8	8.7	1.8	[52]
14	p‐Si	—	0.5 m Na_2_SO_4_	0.5	6	1.1	[53]
15	TiO_2_/CdS/CuInS_2_	Pt	0.1 m Na_2_HPO_4_	1	13	1.82	[51]
16	In_2_S_3_/CuInS_2_	Pt	0.1 m Na_2_HPO_4_	2	18	2.9	[54]
17	BiVO_4_/TiO_2_	—	0.5 m NaHCO_3_	1.5	5.5	1.1	[55]
18	TiO_2_/C‐QDs	Au	0.5 Na_2_SO_4_	—	16	1.89	[56]
D. Poor PEs (STH < 1%)							
19	CuGaSe_2_	Pt	1 m Na_2_SO_4_	—	4.95	0.35	[57]
20	ZnS/CuGaSe_2_	Pt	0.1 m Na_2_SO_4_	—	4.35	0.25	[58]
21	CdS/CuGaSe_2_	Pt	1 m Na_2_SO_4_	5	7.5	0.83	[59]
22	C/CuO	Cu	1 m Na_2_SO_4_	1	3.95	0.56	[45]
23	g‐C_3_N_4_/ZnO	Pt	0.5 m Na_2_SO_4_	5	0.12	0.07	[48]
24	ZnO/ZnFe_2_O_4_	—	0.1 m Na_2_SO_4_	1	2	0.81	[60]

It is worth mentioning some examples of the most studied materials in this field. Despite semiconductors such as GaN, InGaN, or Si‐based^[^ 61, 62, 63 ^]^ are widely involved, they are not the best candidates in PEC WS because they show poor stability, high toxicity, or low availability. Therefore, due to their excellent features, metal oxide semiconductors are more promising as photoelectrode materials. In fact, their electronic properties are easily tunable; they show proper bandgaps and band‐edge positions for solar‐light absorption and water electrolysis, respectively. Furthermore, they can be either intrinsic n‐type (e.g., TiO_2_, ZnO, WO_3_) or p‐type (e.g., Cu_2_O) semiconductors, so they can be employed both as photoanodes and as photocathodes. TiO_2_ has been the most widely explored and promising photocatalytic material for EC and PEC WS, but its bandgap in the UV range (3.0–3.2 eV) can be activated with UV light exclusively, which covers only the 5% of the entire solar spectrum. Other metal oxides semiconductors have been taken into account; an example is WO_3_, which has received attention thanks to its bandgap in the visible range (2.4–2.7 eV) and its band‐edge potentials position. However, one of its main drawbacks is the fast recombination of the photogenerated charge carriers and the poor water oxidation kinetics.^[^ ^28^ ^]^

α‐Fe_2_O_3_ or hematite seems to be a good candidate. It is economic, nontoxic, easily available, and chemically stable against photocorrosion. Thanks to its narrow bandgap (2.1 eV), α‐Fe_2_O_3_ can absorb a wide range of wavelengths of the incident solar spectrum, including visible light. Moreover, some studies reveal that hematite owns high solar‐to‐hydrogen (STH) efficiency (16.8%) and long‐term stability (1000 h). However, iron oxide shows some issues to be addressed such as the small optical absorption, rapid electron–hole recombination, and short carrier diffusion lengths, which have lowered its photocurrent density achieved up to now.^[^ 64, 65 ^]^

Similarly, ZnO has been studied as a promising photoanode due to its peculiar properties: availability, ease of synthesis, efficiency, and suitable band structures for PEC WS. Unfortunately, it suffers from many points of view. Most importantly, only the 4% of solar radiation can be absorbed by ZnO because it is a wide bandgap semiconductor (*E*_g_ ≈ 3.3 eV) and, as a result, it has poor STH efficiency. In addition, ZnO, such as other semiconductors (CdS and Cu_2_O), is highly exposed to photocorrosion.

This review will focus on two of the most promising metal oxide semiconductors, in our opinion, i.e., on α‐Fe_2_O_3_, ZnO, and their composites, with particular attention to dependence of PEC WS efficiency on the morphology of the nanostructures.

The range of light absorption, the photocorrosion resistance, and the efficiency of hydrogen evolution strongly depend on the structure and the morphology of the photocatalyst.^[^ 66, 67 ^]^ For this reason, the focus of this work will be on the comparison of different morphologies of ZnO, α‐Fe_2_O_3_, and their composites to understand how their stability and STH conversion efficiency are affected, and the benefits offered by the combination of the two materials, especially at the nanoscale.

Briefly, in this review, we first describe in detail the most widely used morphologies in the field of PEC WS for ZnO, α‐Fe_2_O_3_, and their composites. Then we compare them to highlight how the combination of ZnO and α‐Fe_2_O_3_ and, most importantly, the morphology can affect efficiencies. Furthermore, one of the main approaches to influence the optical and electrical properties of materials is doping. Through this strategy, the processability can be improved functionalizing the nanomaterials and modifying them at the atomic level. Different metallic and nonmetallic ions can be incorporated into the structures, yielding to changes in the density of charge carries and in the interactions at the material/electrolyte interface. Moreover, increase in the charge separation and reduction of recombination rates can be easily identified as consequences of doping. On the other hand, the presence of impurities can lower the crystallinity, affecting the overall performance of the catalyst and its morphology. An in‐depth discussion on the effects of doping on ZnO, hematite, and ZnO/hematite heterostructures will be led throughout the review, giving to the reader a vision on the potential of doping as strategy for improving the performances of already known materials. Finally, we draft some conclusions emphasizing how engineering the properties, specifically morphology and materials composition, is important to reach an efficient and active PEC WS photoelectrode. Furthermore, our goal is to elucidate why and how ZnO/α‐Fe_2_O_3_ heterojunctions are promising for WS, what are their main merits and limits, and how the present bottlenecks can be overcome.

## 2 Efficiency Standards to Compare Different Materials

The morphology of the photocatalysts has an impact on the PEC WS efficiency. In general, hydrogen evolution is strongly affected by morphology and chemical composition because the absorption and reflection of light and the separation of photoinduced charge carriers influence the photocatalytic decomposition reaction of water.^[^ ^68^ ^]^ For this reason, it is important to clarify the efficiency standards, which are typically used. Efficiencies are parameters to define the performance of PEC WS systems and to make a comparative evaluation among them. The most important ones can be divided in two main classes: benchmark and diagnostic efficiencies. The latters are useful to describe and understand system/interface performance and they are 1) applied bias photon‐to‐current efficiency (ABPE), 2) external quantum efficiency (EQE), equal to incident photon‐to‐current efficiency (IPCE), and 3) internal quantum efficiency (IQE), equal to absorbed photon‐to‐current efficiency (APCE). However, the STH conversion efficiency is the only one that can be used to determine the overall H_2_ production. It should also be used as a standard value to compare PEC candidate materials. STH efficiency describes the real efficiency of a PEC WS system exposed to solar (or simulated solar) irradiance, under zero‐bias conditions. Provided that certain conditions are met,^[^ ^69^ ^]^ the STH standard definition can be expressed by Equation (4), which is based on the direct measurement of the true H_2_ production rate by an analytical method

(6) $$\text{STH}={\left[\frac{\left({\text{mmolH}}_{2}/s\right)\times \left(237000{\text{ J mol}}^{-1}\right)}{P_{\text{total}}\left({\text{mW cm}}^{-2}\right)\times \text{Area}\left({\text{cm}}^{2}\right)}\right]}_{\text{AM}1.5G}$$

Or, alternatively, Equation (7)

(7) $$\text{STH}={\left[\frac{\left|j_{\text{sc}}\left({\text{mA cm}}^{-2}\right)\right|\times \left(1.23V\right)\times \eta_{F}}{P_{\text{total}}\left({\text{mW cm}}^{-2}\right)}\right]}_{\text{AM}1.5G}$$

where the output power produced by the device is calculated as the product of the short circuit photocurrent density (*j*_sc_), the voltage (1.23 V referred to the thermodynamic WS potential), and the Faradaic efficiency for hydrogen evolution (*η*_F_). *P*_total_ is the total integrated power input density of the incident light under standard air mass 1.5 global (AM1.5G) irradiation (100 mW cm^−2^).

Several factors may affect the overall efficiency and performance of PEC WS: 1) light absorption (efficiency labeled as *η*_A_), 2) charge separation (*η*_CS_), 3) charge transport (*η*_CT_), and 4) charge collection/reaction efficiency (*η*_CR_). The incorporation of such variables allows to express the STH conversion efficiency as^[^ ^69^ ^]^

(8) $$\eta_{\text{STH}}=\eta_{A}\times \eta_{\text{CS}}\times \eta_{\text{CT}}\times \eta_{\text{CR}}$$

STH efficiency is the only one that can be considered as a benchmark for the comparison of different materials. Furthermore, high values obtained from the diagnostic efficiencies do not necessarily mean high STH efficiency values. However, it becomes challenging to compare STH efficiencies. In fact, in the literature several standards, which are used for this kind of measurement, can be found. They cannot be compared because they are carried out under different experimental conditions. For these reasons, in this review we will not consider only STH efficiency. As ABPE is not a genuine STH measurement, because it requires an applied bias that generally increases the current, we will focus on IPCE or EQE. It describes the photocurrent generated per incident photon as a function of the light wavelength. Ideally, under zero‐bias conditions, it is possible to estimate the maximum STH efficiency by integrating IPCE over the entire solar spectrum. In this context, IPCE describes the maximum value of possible efficiency the incident light can produce H_2_, assuming that all electrons are used for H_2_ evolution (and holes for O_2_ evolution), instead of by‐products or corrosion. The latter assumption is not always true, hence it is important to keep in mind that the efficiency measurement can be overestimated when the Faradaic efficiency is unknown.

IPCE can be expressed by Equation (9),^[^ ^70^ ^]^ as the ratio between photocurrent converted to electron rate and the incident photons rate converted from a calibrated power of the monochromatic light source.

(9) $$\text{IPCE }(\lambda )=\text{EQE }(\lambda )=\frac{\text{electrons}/({\text{cm}}^{2}{\text{ s}}^{-1})}{\text{photons}/({\text{cm}}^{2}{\text{ s}}^{-1})}=\frac{\left|j_{\text{ph}}\left({\text{mA cm}}^{-2}\right)\right|\times 1239.8\left(V\times \text{nm}\right)}{P_{\text{mono}}\left({\text{mW cm}}^{-2}\right)\times \lambda \left(\text{nm}\right)}$$

where 1239.8 (V nm) is the product of the Planck's constant (*h*) and the velocity of light (*c*), *P*_mono_ is the calibrated and monochromatic light source power intensity in mW cm^−2^, and *λ* (nm) is the wavelength at which this power is measured.

Most of the studies report only the values of the photocurrent density, which is indeed important, but not always sufficient for a critical analysis of the experiments.

## 3 ZnO and α‐Fe_2_O_3_: Two Good Candidates

Among all metal oxide semiconductors, which have been extensively studied for sustainable applications such as energy conversion, two important ones are ZnO and α‐Fe_2_O_3_. These metal oxides have attracted much attention since the early 20th century, not only because of their optical and electronic properties, nontoxicity, earth‐abundance, and cheapness, but also for their broad chemistry leading to many synthesis strategies, which is an industrially important aspect for future applications.

Hematite (α‐Fe_2_O_3_) has historically attracted great interest as a photoanode material thanks to its relatively narrow bandgap (2.0–2.2 eV), enabling the absorption of 40% of the solar spectrum and the suitable position of its VB.^[^ ^71^ ^]^ Furthermore, it fulfills many of the requirements for photoelectrode materials for PEC because it is stable, nontoxic, cheap, and abundant. Its theoretical photocurrent density is 12 mA cm^−2^ (with a STH efficiency of 15.4%), which it is still not reached by hematite photoanodes. In fact, the PEC performance is limited by a low electron mobility (≈10^−2^ cm^2^ V^−1^ s^−1^), short hole diffusion length of 2–4 nm compared to its light penetration depth (≈120 nm at *λ* = 550 nm), and very short excited‐state lifetime of ≈10 ps.^[^ 72, 73 ^]^ Additionally, the CB of α‐Fe_2_O_3_ lies ≈0.4 V below the H^+^/H_2_ redox potential, thus suggesting that a bias has to be applied for hydrogen reduction.

Regarding ZnO, usually researchers perform water electrolysis in extremely acidic electrolytes with inert anions (e.g., sulfate and perchlorate) or in extremely basic electrolytes (e.g., KOH and NaOH). The contact between ZnO and the electrolyte generates a charge transfer at the photoanode/electrolyte junction. This phenomenon takes place because of a difference in the position between ZnO's Fermi level and the potential of the redox pair of the electrolyte. Subsequently, the charge distribution at the interfaces changes according to the charge transfer. In the process, an electric field seems to form due to the bending of the edges, whose behavior depends on the difference between the electrolyte potential and the Fermi level. The so‐generated charge carriers can be easily separated under the aforementioned electric field at the interface between the solid (ZnO) and the liquid (electrolyte). This allows for reduction and oxidation reactions, which are the basis for the WS process.^[^ ^74^ ^]^ However, the ZnO direct wide bandgap (3.37 eV) does not allow to use it as efficient solar light harvester. Another important issue is its exposure to photocorrosion, which impairs its stability over time.

Tuning the nano‐ and mesoscale structures of both ZnO and α‐Fe_2_O_3_, to extend their applications, is one of the main goals for nanotechnology. Thus, engineering and designing new nanostructures with the possibility of tuning dimension, size, porosity, crystal facets, and mesoscale architectures have achieved great importance in the improvement of their performances in various applications, including PEC WS. In summary, ZnO is a wide bandgap semiconductor, unable to naturally absorb visible light, unless doping or other sensitization strategies are applied. α‐Fe_2_O_3_ exhibits ideal bandgap for visible light absorption, but its low charge mobility hampered its successful application. However, as recently demonstrated, the combination of ZnO and α‐Fe_2_O_3_ may result in improved overall functionality, which may open new perspectives for successful exploitation of these “old” materials in PEC WS.

## 4 Morphology

**Scheme 1:**
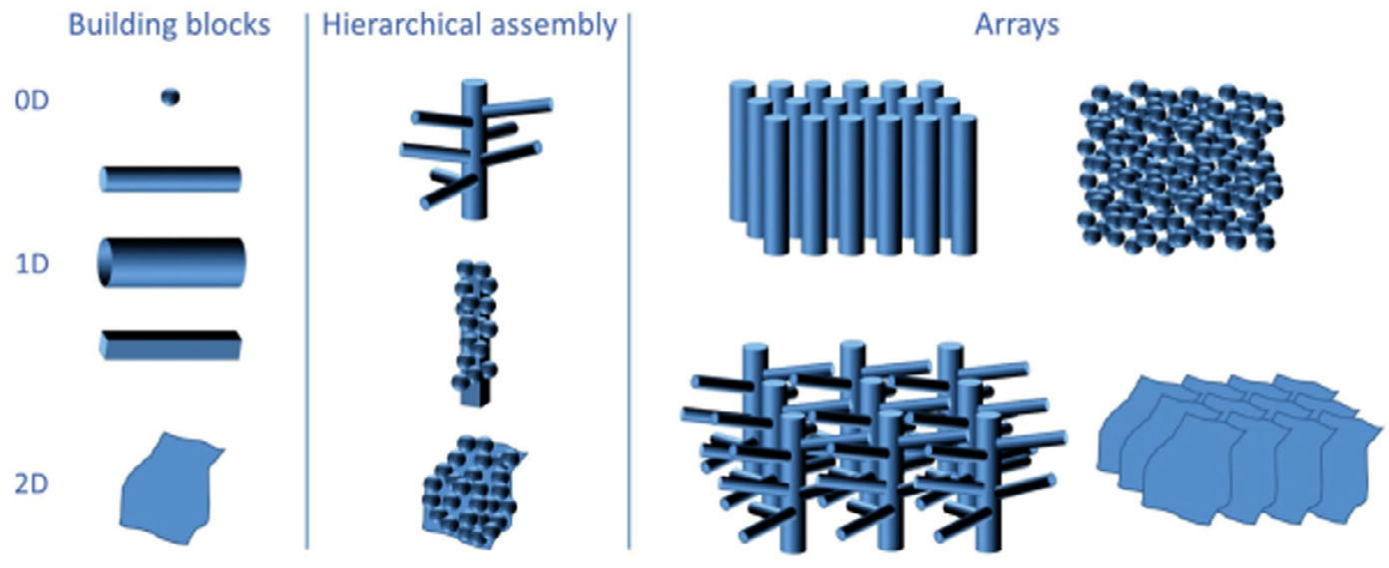
The most commonly produced 0D–2D structures (NPs, NWs, nanotubes [NTs], NRs, nanosheets [NSs]) reported in the literature as building blocks to fabricate hierarchically assembled geometries and/or nano/microarrays. Reproduced with permission.^[^ ^76^ ^]^ Copyright 2017, Wiley‐VCH.

Many examples of nanostructures, including variously shaped nanoparticles (NPs), nanowires (NWs), and nanorods (NRs) (**Scheme** **1**), can be prepared using different deposition methods or chemical and physical synthetic strategies for both ZnO and α‐Fe_2_O_3_.^[^ ^75^ ^]^

In several cases photoelectrochemically active metal oxides, such as hematite and ZnO, are grown or deposited on conductive glass substrates (i.e., fluorine‐doped tin oxide [FTO] glasses or indium tin oxide [ITO]). One of the main drawbacks of this strategy is that charge collection through the conductive substrates may contribute to recombination processes.^[^ ^76^ ^]^ Consequently, accurate photoelectrode design has become more and more important. Several applications of hierarchical and complex nanostructures based on ZnO and hematite have emerged. In fact, changes in the morphology (from OD, to 1D, 2D, and 3D structures) influence the surface‐area‐to‐volume, the electron transport velocity, the charge separation efficiency, and the charge recombination rate. The possibility of modifying such characteristics makes ZnO‐ and hematite‐based nanomaterials suitable for photoelectronic applications, as hydrogen and oxygen generation, and photocatalysis and photoelectrocatalysis.

In this review, many reported performances are related to changes in the morphology. This is done to highlight the importance of such parameter and to make the reader conscious about the potentiality of this modification strategy to achieve important results in WS.

For this purpose, the following article discusses the most peculiar and widely used morphologies of materials based on ZnO, α‐Fe_2_O_3_, and their composites, which have been reported recently in different studies.

### 4.1 ZnO

ZnO is an n‐type semiconductor^[^ ^77^ ^]^ that is getting a lot of attention due to several outstanding properties such as nontoxicity,^[^ ^78^ ^]^ low cost, high thermal conductivity,^[^ ^79^ ^]^ and a wide direct bandgap (3.37 eV).^[^ 80, 81 ^]^ The shrinking of such material at the nanoscale modifies the properties of the material itself, changing its electronic structure, the surface‐to‐volume ratio, the density of defects,^[^ ^82^ ^]^ and so on, therefore expanding its range of applications.^[^ ^83^ ^]^ One of the possible applications is photoelectrocatalysis. In this field, the morphology and the size play an important role in the overall performance.

Several synthetic routes have been developed to produce ZnO with almost all the possible morphologies, including 0D, 1D, 2D, and 3D shapes.

#### 4.1.1 0D Nanostructures: QDs

Obtaining quantum confinement effects in 0D structures of wide bandgap semiconductors is a challenge, due to the very small dimensions required to reach quantum confinement. However, it is worth mentioning an example for the fabrication of chemically bonded BiVO_4_/ZnO quantum dot (QD) heterostructures. The process involves the dispersion of the ZnO QDs in a BiVO_4_ precursor solution. After the calcination process, the ZnO QDs are uniformly distributed within the BiVO_4_ nanocrystals and at their surface, originating a heterostructure, which is applied for PEC WS.

From IPCE spectra, it can be observed that these chemically bonded interfaces are able to operate in the UV region as well as in the visible region. In fact, the presence of ZnO QDs at the surface is demonstrated to give a contribution to the photocurrent density, enhancing it and allowing to reach 5.5 mA cm^−2^ (1.23 V vs reversible hydrogen reaction (RHE)) without any need of a cocatalyst.^[^ ^84^ ^]^

#### 4.1.2 1D Nanostructures: NRs, NWs, Caterpillar, and Nanobelts

A wide range of approaches to fabricate ZnO NRs have been proposed, mainly based on hydrothermal synthesis.

A lukewarm hydrothermal method results in the growth of ultralong 1D ZnO NR arrays onto FTO glass substrate at 65 °C. These ≈10 μm‐long 1D ZnO NRs show a photocurrent response of ≈0.9 mA cm^−2^ at 1 V_Ag/AgCl_ and an IPCE value that is 4 times higher with respect to the ≈4 μm‐long counterpart at a wavelength below 400 nm, as a result of an enhanced light harvesting ability. The characterization revealed the presence of several crystal defects that were creating midgap states and leading an appreciable increase in the specific surface area. The combination of these factors allowed these nanostructures to harvest light in the visible range in addition to the UV one, expanding the absorption spectrum.^[^ ^85^ ^]^

ZnO NRs are fabricated on preseeded ITO glass substrate through hydrothermal synthesis. According to different precursor solution concentrations, the highest photocurrent density is equal to 0.483 mA cm^−2^ and shows a photoconversion efficiency (PCE) of 0.56% (precursor solution 0.03 m at 100 °C growth temperature) due to the improvement in orientation of the rods and in enhancement of the light harvesting.^[^ ^86^ ^]^

To further improve the PEC WS ability of ZnO NR arrays obtained through hydrothermal synthesis, a simple approach was proposed. A vacuum annealing at high temperature was tested to annihilate the Zn(OH)_2_ species formed during crystal growth, and acting as trapping sites for photoexcited charges. The treated nanostructures show a photocurrent density of ≈600 μA cm^−2^ at a potential of 0.57 V_SCE_ (1.23 V_RHE_, V_RHE_  = V_SCE_ + 0.0591 × pH + 0.244). This result can be attributed to the generation of oxygen vacancies in the ZnO NRs. IPCE values below 400 nm increased according to the increasing annealing temperature. Passivation of trap states, in fact, induces an increase in the charge carrier density and a more efficient charge separation.^[^ ^87^ ^]^

Outstanding results can also be obtained at lower annealing temperatures, by decorating ZnO NRs with optically active nanocrystals. ZnO NRs/CdS QDs heterojunction were fabricated by atomic layer deposition, hydrothermal method, and ionic layer adsorption–reaction method and thermally treated via a two‐step low‐temperature process. The photocurrent density reaches 9.16 mA cm^−2^ at 0.4 V_SCE_ and the IPCE is as high as 95% at 350 nm, meaning that the two‐step thermal treatment improves the charge transfer kinetics and the efficiency in separating the photogenerated carriers across the ZnO/CdS heterostructure.^[^ ^88^ ^]^

Another way to improve PEC performances is to fabricate ZnO/CdS/CdSe heterostructures on Zn foil substrate using a three‐step method that consists of hydrothermal growth, successive ionic layer adsorption and reaction (SILAR), and modified chemical bath deposition (CDB). In fact, this heterostructure shows a photocurrent density of 6.244 mA cm^−2^ at −0.2 V versus Ag/AgCl, a PCE of 4.381% under light illumination of 100 mW cm^−2^, and a relevant maximum IPCE value of 81% at 400 nm thanks to improved charge separation and transfer.^[^ ^89^ ^]^

Doping of ZnO nanostructures to modulate their optical and/or electrical properties is an alternative way to enhance their PEC properties. Incorporation of Y ions into vertically aligned ZnO NR arrays causes a change in the optical and electrical properties of the NRs themselves. In fact, the bandgap energy was gradually decreased according to different mole percentage, letting photocurrent density to reach values of 1.2 mA cm^−2^ at 0.2 V versus Ag/AgCl, a value that is 3 times higher than the nondoped structures. As the concentration of yttrium ions was increased up to 1.2 mol%, the photocurrent density of the samples started to decrease suggesting that high doping concentration created high levels of defects, which were acting as recombination centers, leading to an overall reduction of the photocatalytic activity. These results are justified by the incorporation of the Y^3+^ ions into the NRs lattice. The doping process forms new energy levels within the ZnO bandgap structure of the undoped material, and this gives a relevant contribution in preventing electron–hole pair recombination.^[^ ^90^ ^]^

Similar results are obtained by incorporating Ge ions into ZnO NRs. The presence of Ge dopant allows to reach photocurrent density values of 12 mA cm^−2^ at 1.654 V_RHE_. The PCE of the fabricated Ge‐doped ZnO NRs was calculated according to the Equation (10)

(10) $$\eta (\%)=\frac{\left(\text{total power output}-\text{electrical power input}\right)}{\text{light power input}}\times 100=j_{p}[\frac{E_{\text{rev}}^{0}-\left|E_{\text{appl}}\right|}{I_{0}}]\times 100$$

where *η* represents the PCE, *j*_p_ is the photocurrent density (mA cm^−2^), $\left|E_{\text{appl}}\right|$ is the applied potential, $E_{\text{rev}}^{0}$ is the standard reversible potential, and *I*_0_ is the incident power density (100 mW cm^−2^). The so‐obtained Ge‐doped ZnO NRs exhibited a PCE of ≈3.6% under AM1.5G illumination, which was much higher than what reported in the literature, indicating the suppression of recombination centers upon illumination. It is also shown a maximum IPCE peak (54%) at ≈405 nm, a value that is much higher than the ones reported for pristine ZnO.^[^ ^91^ ^]^

These structures have always been prepared on conducting substrates such as ITO and FTO, due to their superior transparency and conductivity. But they also suffer from limited success due to their expensive, fragile, and brittle nature. The processing of ZnO at low temperatures allows for its application onto flexible substrates, relaxing in this way the operating conditions.^[^ ^92^ ^]^ Therefore, it is interesting the development of strategies for processing ZnO that overcome the temperature limit and that ensure good results as well. It is reported a case, in which ZnO NR‐based photoanodes are prepared on a flexible micropatterned and on a flat polyurethane acrylate (PUA) substrate. The photocurrent densities generated by the flat and the micropatterned substrates are shown to be ≈0.17 and ≈0.52 mA cm^−2^ at 1.23 V versus RHE, respectively, while the IPCE values at a wavelength of 400 nm are reported to be ≈3.8% for the flat PUA substrate, whereas for the micropatterned substrate the IPCE value is ≈1.4 times higher. The enhanced PEC performances were attributed to an improvement in charge collection and transport capability and high surface area owing to micropillar structure.^[^ ^93^ ^]^

In addition to NRs, NW morphology with significantly increased aspect ratio is also very important. NWs are one of the most studied nanostructures. Diverse methods have been proposed for their fabrication.

One novel strategy is to play with hydrodynamic conditions during anodization, promoting the formation of ordered ZnO NWs, and influencing the photocurrent density and its stability. The anodization is carried out in a two‐electrode cell configuration under different hydrodynamic conditions. Previous to anodization, Zn rods of 8 mm diameter are treated and abraded, and then anodized in a 50 mM NaHCO_3_ electrolyte at a potential of 10 V for 10 min at different rotation speeds. The ZnO nanostructures present a high photocurrent density response during WS experiments, especially for the sample that has been anodized at 5000 rpm, which presents an improvement in the photocurrent density of ≈150% compared to the sample anodized under stagnant conditions. At higher rotation speeds, the growth of the NWs is slower and consequently more organized and homogeneous, in contrast with what was obtained under stagnant conditions. This hydrodynamic parameter influences the growth by 1) removing dissolved zinc near the electrode surface, decreasing the NWs growth rate by precipitation, and 2) improving the mass transfer toward the electrode surface, promoting the presence of soluble species accounting for the NWs formation.^[^ ^94^ ^]^

Similarly, ZnO NWs can be obtained by simple anodization in different aqueous bicarbonate electrolytes. The highest values for photocurrent density are 0.34 and 0.29 mA cm^−2^ at −0.46 V_Ag/AgCl_ under simulated solar light in electrolytes containing 10% v/v ethanol and 25% v/v glycerol, respectively. This study demonstrated the strong influence on the morphology of the addition of ethanol and glycerol to the bicarbonate electrolyte used for the anodization. An increase in the amount of ethanol implied a decrease in the growth rate of the nanostructures and a shortening in the length of the so‐formed NWs, while glycerol containing electrolytes produced new morphologies such as NTs, nanospheres, and a compact nanosponge.^[^ ^95^ ^]^

Other possible strategies involve doping and coating of NWs with an external shell. As already mentioned for ZnO NRs, doping can alter the optoelectronic properties also in ZnO NWs, tuning both optical absorption and electronic transport. Sb‐doped p‐type ZnO NWs were obtained through a low‐temperature hydrothermal method. Comparing the photocurrent density of Sb‐doped and undoped, it is possible to observe that it increases as the Sb‐doping concentration rises up to 0.2%, while, above this value, it starts to decrease with increasing doping concentration. After annealing, the 0.2 Sb/ZnO sample shows a photocurrent density of −0.85 mA cm^−2^ at 0 V_RHE_, a value that is ≈17 times larger than the n‐ZnO NWs under simulated solar sun light. The presence of Sb impurities improves charge transport and accelerates charge separation.^[^ ^96^ ^]^

A methodology is developed to dope ZnO NWs with S ions through a one‐step solution method via direct reaction of zinc foil in an aqueous solution of sodium hyposulfite at constant temperature. Half‐arc mesoporous super‐structured NWs were fabricated. The photocurrent density was 0.5 mA cm^−2^ and the power conversion efficiency was measured to be 0.44% from 500 to 300 nm, leading the way for a new doping‐based approach to enhance the photochemical properties of ZnO NWs. In fact, it was demonstrated that elemental sulfur doping can reduce the bandgap for ZnO, thereby extending the light absorption range and increasing the separation of charges.^[^ ^97^ ^]^

It was demonstrated that coating promotes PEC properties as well because the design of a semiconductor heterostructure effectively promotes a fast charge separation and a slow recombination of electron–hole pairs, in addition to add anticorrosive properties.^[^ ^98^ ^]^ In fact, metal oxide coatings are broadly employed for anticorrosion due to their inexpensive manufacture and superior performances in the anticipation of corrosion. In a recent research, it is reported a mixed‐metal organic framework‐coated ZnO NW array (ZnNi MOF@ZnO) that exhibits a high photocurrent density of 1.40 mA cm^−2^.^[^ ^99^ ^]^ A Fe_2_O_3_ coating enhances the PEC WS ability of ZnO/Fe_2_O_3_ core–shell NWs with respect to the bare ZnO NWs counterpart. The weak diffraction intensity of the Fe_2_O_3_ reveals the deposition of a thin layer of α‐Fe_2_O_3_ with a thickness of a few nm. The presence of a Fe_2_O_3_ shell decreases the defect distribution on the surface, thus decreasing the density of recombination centers. The ZnO/Fe_2_O_3_ core–shell heterojunction interfacial charge transfer reduces the loss of photocatalytic efficiency that arises from a reduction of electron–hole recombination. Furthermore, the presence of the Fe_2_O_3_ thin layer allows for sensitization of the ZnO NWs to visible light, improving the light harvesting ability of the material.^[^ ^100^ ^]^

**Figure 2:**
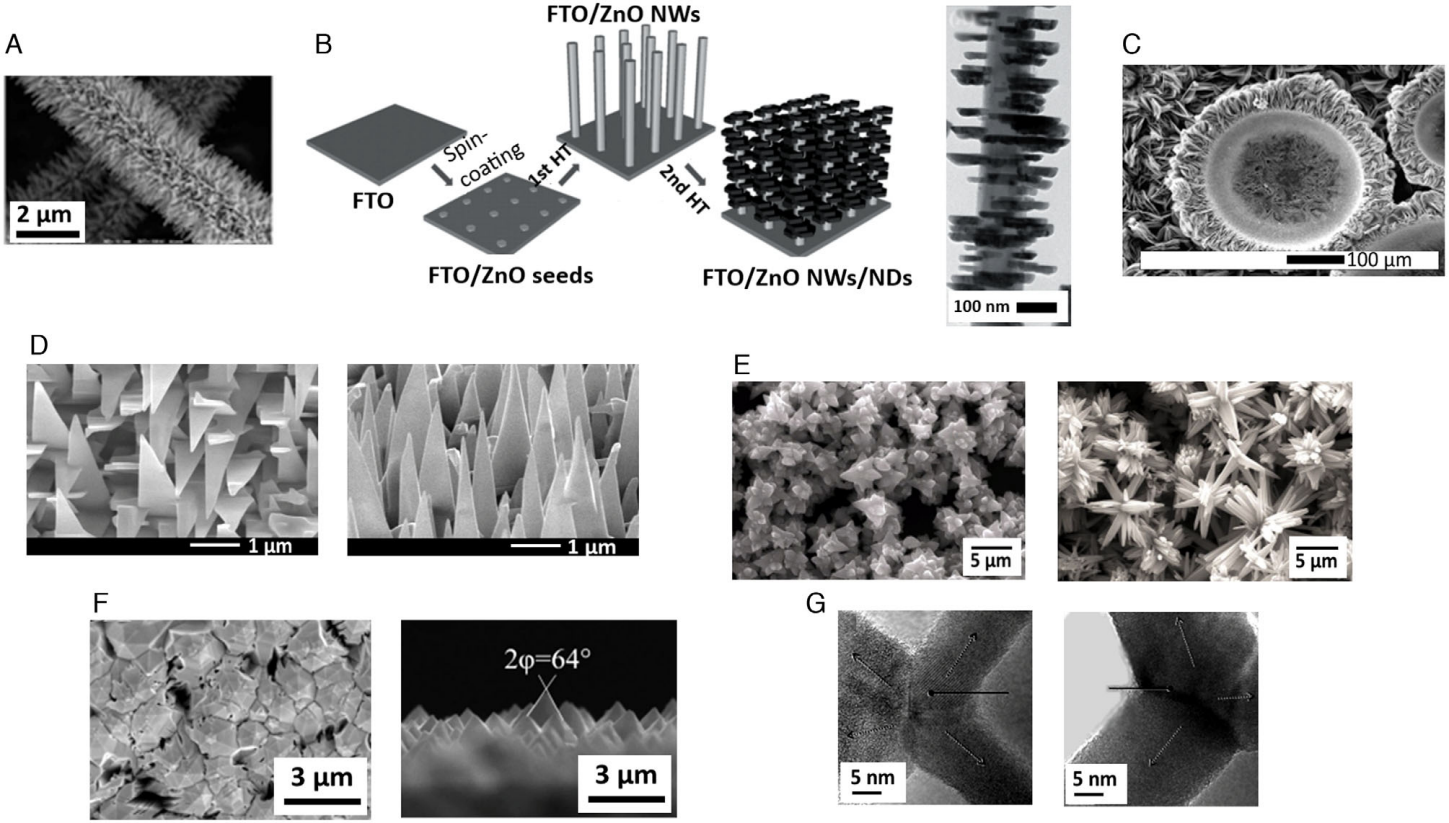
A) High‐magnification scanning electron microscopy (SEM) image of “caterpillar‐like” BZNs. Reproduced with permission.^[^ ^101^ ^]^ Copyright 2014, American Chemical Society; B) schematic illustrating the preparation processes of ZnO NWs/NDs (left) and transmission electron microscopy (TEM) images of ZnO NWs/NDs (right). Reproduced with permission.^[^ ^108^ ^]^ Copyright 2014, Wiley‐VCH; C) SEM image of zinc after anodization in 0.2 m (NH_4_)_2_SO_4_ + 0.2 m NH_4_Cl at 1 V, 40 min. Reproduced with permission.^[^ ^105^ ^]^ Copyright 2011, Elsevier; D) top‐view and 45° tilted SEM images of ZnO nanosail arrays. Reproduced with permission.^[^ ^109^ ^]^ Copyright 2021, Wiley‐VCH; E) Field‐emission scanning electron microscopy (FESEM) images of star‐like ZnO nanostructures (left) and flower‐like ZnO nanostructures (right). Reproduced with permission.^[^ ^110^ ^]^ Copyright 2019, Elsevier; F) top‐view and cross‐sectional SEM images of pyramidal ZnO. Reproduced with permission.^[^ ^111^ ^]^ Copyright 2021, Elsevier; G) High‐resolution transmission electron microscopy (HRTEM) images illustrating four leg joint regions in small diameter tetrapods (left) and large diameter tetrapods (right). Reproduced with permission.^[^ ^112^ ^]^ Copyright 2021, American Chemical Society.

It is also reported the procedure and the PEC WS applicability of another very interesting 1D nanostructure: the caterpillar‐like ZnO nanostructures (**Figure** **2**A). These novel branched ZnO nanofibers have been proved to have enhanced PEC properties compared to parental ZnO nanofibers and vertically aligned NW arrays. They showed a maximum current density of 0.524 mA cm^−2^ at 1.2 V versus Ag/AgCl. Such value is 150% higher than that of ZnO NW arrays. The maximum efficiency was observed to be ≈0.165% (at 0.89 V vs RHE), that is, ≈150% higher than the vertically aligned NW counterpart. Additionally, no photocurrent density saturation was observed on these nanostructures at more positive potentials (up to +1.2 V), implying an enhanced harvesting of light and a higher efficiency in electron–hole separation. In fact, the fine branches with their high length‐to‐diameter aspect ratios facilitate the charge separation and hole diffusion. The combination of high aspect ratios, charge collection, and separation yields into remarkable performances.^[^ ^101^ ^]^

Indium‐doped ZnO nanobelts can be obtained through chemical vapor deposition (CVD) using a SiO_2_/Si substrate with gold thin film as a catalyst. The presence of indium is necessary to improve the resistance of this nanostructure to corrosion: the self‐corrosion potential increased to 0.172 V thanks to the presence of In, while the self‐corrosion current density decreased to 3.16 nA cm^−2^, yielding into an overall reduced corrosion rate in the electrochemical solution (HCl, pH 6). Electrical properties have been analyzed and the current has been shown to be kept about ±15 mA after 43 h corrosion at a bias voltage of ±1 V, indicating a stable transport of electrons under such an electrochemical environment.^[^ ^102^ ^]^

#### 4.1.3 2D Nanostructures: NSs, Sunflowers, Nanodisks, and Nanosails

2D nanostructures have been investigated as well. Few studies exist concerning 2D ZnO NSs, even if 2D nanostructures are very promising for application in photoelectrocatalysis. Several methods such as vapor deposition, hydrothermal synthesis, and anodization could be employed to fabricate such structures.^[^ ^103^ ^]^ A method that takes advantage of hydrothermal synthesis to form ZnO NSs anchored to sodium bismuth sulfide nanoribbons has been proposed.^[^ ^104^ ^]^ Through this method it is possible to obtain ultrasmall ZnO NSs having a hexagonal structure of 30–50 nm wide and their deposition onto the NaBiS_2_ nanoribbons has been proved to work well in both tetracycline degradation and PEC water oxidation using solar radiation. The formation of NaBiS_2_‐ZnO (NSZO) nanocomposite has influenced the bandgap, remarkably changing it. The bandgap of NSZO was estimated to be 1.36 eV and such modification arises from the band bending at the junction of the two semiconductors. Enhanced catalytic activity of NSZO could be attributed to the separation of photogenerated electron–hole pairs and to the generation of a heterostructure, which improves the charge carrier transport. The combination of bandgap engineering and improved light harvesting led to an overall higher photocurrent. The difference in the photocurrent density in the absence and presence of light demonstrates that the nanocomposite is more efficient with respect to the pure samples. This difference is shown to be 13 times higher than ZnO and ≈7 times higher than NaBiS_2_.

Approaching the synthesis through anodization method, it is possible to observe that the concentration of the electrolytes and the applied voltages influence the formation of NSs, and from a current density point of view, it is observed a rapid current decay that then turns to slow gradually, suggesting the formation of oxides at the surface of the zinc foil. The rapid decay can be associated with a rapid raise in the resistance implied by the fast anodization rate.^[^ ^105^ ^]^

According to the increasing anodization time, it is possible also to observe the formation of a sunflower microstructure with a diameter of 400–500 μm, maintaining the same composition of the NSs, but with a higher density at the center (Figure 2C).

An alternative way to manipulate optical and/or electrical properties of 2D ZnO nanostructures is to involve doping. Through an appropriate introduction of impurities, it is possible to tune the bandgap and regulate the charge carrier density. Via a two‐step electrodeposition process, ZnO nanoarrays and ZnO:Cr NSs can be synthesized on F‐doped tin oxide film in order to obtain a ZnO/ZnO:Cr junction. The photocurrent densities are 0.03 mA cm^−2^ for the ZnO NRs, 0.435 mA cm^−2^ for the ZnO/ZnO:Cr structure, and 0 mA cm^−2^ for ZnO:Cr NSs. The comparison with ZnO NRs alone or ZnO:Cr NSs highlighted that this hybrid system exhibited higher PEC performances due to the enhancement in visible light absorption and due to a stronger charge carrier separation. In the same research, ZnO NR–NS structures were integrated with CdS QDs. The introduction of 1D nanostructures enhances the light harvesting and yields a large photocurrent density (4.65 mA cm^−2^ at 0.4 V vs Ag/AgCl) and a high PCE.^[^ ^106^ ^]^

Vanadium‐doped ZnO NSs can be used as photoanode, as well. In this case, the internal dipole polarization affects positively the water oxidation performances. The photocurrent density of positively poled V‐doped ZnO NSs under illumination is 35 mA cm^−2^, a value that in comparison to nonpoled (17 mA cm^−2^) and negatively poled (14 mA cm^−2^) is 100% and 160% higher (all at 1.23 V vs RHE), respectively. This enhancement in the photocurrent density was achieved thanks to a more favorable band bending at the PEC electrode/water interface.^[^ ^107^ ^]^

An interesting approach for enhancing electrochemical properties of ZnO‐based nanomaterials is to functionalize ZnO NWs with ZnO nanodisks (NDs). This composite structure can be obtained through a two‐step hydrothermal method that involves ZnO seeding on FTO glass immersed in a first hydrothermal solution and growth of ZnO NDs immersing the previously obtained NWs in a second hydrothermal solution, as shown in Figure 2B.

The PEC performances of this nanostructure were compared to bare ZnO NWs, showing a significantly higher photocurrent density, a more efficient charge separation, and a reduced recombination. Furthermore, a negative shift of the photocurrent onset potential was observed, indicating that a lower external potential is needed to achieve WS.

IPCE spectra are measured at 0.0 V versus Ag/AgCl and it can be observed that the IPCE value for the composite structure is higher than bare ZnO NWs in the same wavelength region. In detail, the IPCE value for NWs/NDs is about 5.35% at 380 nm, while the IPCE value for NWs is about 0.38%, allowing to conclude that NWs/NDs exhibit a superior PCE than NWs thanks to an enlargement of about 66% in the surface area and to an increased light usage.

Semiconductors with a wide bandgap such as ZnO are not able to absorb visible light and so the presence of a second material to expand the range of absorption is necessary. Similar to what reported before for 1D shapes, an example is reported on sensitization of complex ZnO shapes through deposition of CdS and CdSe QDs to improve the harvesting of light and to expand the absorption in the visible range. With this configuration, a synergistic effect was obtained, with the QDs responsible for the absorption of light, while the ZnO nanostructures were involved in the transport of electrons. According to this strategy the IPCE spectrum recorded at 0.0 V versus Ag/AgCl shows that the efficiency is improved and approaches 80% in the range of 475–525 nm. The photocurrent density was measured to be 12.8 mA cm^−2^, confirming that cosensitizing ZnO NWs/NDs with CdS and CdSe further improves the PEC performances.^[^ ^108^ ^]^

ZnO can present itself in four low‐index surfaces named nonpolar m‐plane (1010) and a‐plane (1120), and polar c‐plane (0001)‐Zn and (0001)‐O surfaces. It is possible to grow epitaxially well‐aligned nonpolar (1120) ZnO nanosail arrays (Figure 2D) on LGO (010) substrate via chemical vapor deposition by regulating the Au catalyst thickness, growth temperature, and gas flow rate. The photocurrent density of a‐nanosails was measured to be 0.62 mA cm^−2^ at 0.2 V bias, indicating the potential application in PEC. It is also demonstrated that the maximum PCE of these a‐nanosails is 0.16%.

The enhancement in the photocurrent and PEC performances could be ascribed mainly to a stronger absorption of light, a slower carrier recombination, and a fastening in the electron transport. The large surface area allows for a larger harvesting of the light radiation and it influences the capture of charge carriers for WS reactions. This can be further demonstrated by the higher double‐layer capacitance value of the nanosails (0.52 mF cm^−2^), which promotes the diffusion of adsorbed species and increases the availability of active sites. At the same time, the large surface‐to‐volume ratio enhances the process of charge transfer from the interface to the electrolyte. Another interesting feature is the single crystal nature of this nanostructure, which contributes to a more efficient charge transport and reduces the charge carrier recombination.^[^ ^109^ ^]^

#### 4.1.4 3D Nanostructures: Nanoflowers, Nanostars, Nanopyramids, and Tetrapods

Among 3D structures, nanoflowers and nanostars must be mentioned (Figure 2E). Different morphologies were reported to be obtained in a one‐step hydrothermal method at different pH levels of Zn^2+^ ion complex solution. The structures showed a significant photoresponse enhancement for nanoflowers and nanostars with a photocurrent density of 0.34 and 0.31 mA cm^−2^ at overlapping equilibrium potential of oxygen (1.23 V vs RHE) and hydrogen evolution (0 V vs RHE), respectively. This can be explained by the lack of defects in the single crystalline branched nanostructure that significantly enhances its efficiency by uplifting charge separation and/or transport, along with its high surface area. In addition, no photocurrent saturation was recorded at more positive potential, confirming the efficient charge separation upon illumination.^[^ ^110^ ^]^

Another interesting 3D morphology is nanopyramidal structures (Figure 2F). Different methods have been proposed for this purpose (e.g., hydrothermal reactions): a new approach involves the CVD growth of crystalline ZnO pyramidal arrays on a transparent conductive substrate. The latter is needed to minimize the interfacial electrical resistance, yielding an overall more robust and more manageable material. Such morphology shows a higher photocurrent density under UV light compared to ZnO NRs. In fact, the nanopyramidal ZnO generates a current density of 0.45 mA cm^−2^, a substantial improvement with respect to the current density of 0.26 mA cm^−2^ produced by ZnO NRs (at 0.6 V vs Ag/AgCl reference electrode). The comparison in their experimental current densities (a steeper slope in the linear region before the saturation area of the nanopyramidal ZnO can be observed) indicated that the photogenerated charge carriers were more effectively separated in the nanopyramidal ZnO nanostructures. Moreover, the active surface area of pyramidal ZnO was larger than that of the NR structure. Hence, a lower charge transfer resistance and a larger surface area were the main reasons for improved performances of the ZnO nanopyramids.^[^ ^111^ ^]^

Via combustion method it is possible to obtain novel 3D nanostructures based on ZnO, such as tetrapods that consist of four connected NRs (Figure 2G). The outstanding characteristic of tetrapods is that they are made of legs that branch radially from the central region in such a way that on a flat surface one leg is always vertical while the other legs are pointing downward. The 1D legs arrangement of these tetrapods leads to an improvement in the electrochemical properties because of the enhanced electron transport, as demonstrated by the measured resistivity (10^−2^ W m). Other ZnO nanostructures show one order of magnitude higher resistivity, demonstrating that this precise morphology provides an efficient direct pathway for electron conduction. Different sizes can be achieved using the same experimental conditions of temperature, synthesis, and so on and as the method does not affect their production, it is possible to study how their morphology affects the electrochemical properties. In fact, compared with ZnO NRs and NPs, ZnO tetrapods demonstrated better ones. The larger tetrapods (diameter of 120 nm) showed the highest active surface value, and therefore a higher active area was connected to a higher electron transfer rate.^[^ ^112^ ^]^

### 4.2 α‐Fe_2_O_3_

Strategies to maximize light absorption and minimize carrier recombination losses are taken into account in order to overcome α‐Fe_2_O_3_ limitations, including low electron mobility, short hole diffusion length, and very short excited‐state lifetime, as mentioned before. Nonoptimized morphology and structure of photoelectrodes lead to abundant grain boundaries or defects and lack of directional charge transfer to the back contact, which, in turn, cause low electron mobility and charge recombination, and therefore low solar‐to‐fuel production efficiency. On the contrary, morphological and structural optimization of hematite nanocrystals has proved to significantly increase the photocurrent density.^[^ ^113^ ^]^

For the purpose, it is interesting to consider 1D nanostructures such as NRs and NWs, which have been extensively studied, and 3D nanostructures such as nanocubes (NCs). To elucidate how different morphologies affect the PEC performance, hematite samples with the cited shapes can be grown by hydrothermal method on FTO substrate and doped with Sn. X‐ray diffraction (XRD) patterns suggest an oriented growth on the (110) plane for NRs; compared to NRs, NWs exhibit an increased diffraction intensity of the (110) plane, thus indicating a more highly oriented growth in this direction than NRs. In the case of NCs, oriented growth on both (110) and (104) planes can be observed.

NWs show a thinner film thickness with respect to NR, thus leading to a slightly lower light absorbance but a higher carrier density. They also have a higher conductivity thanks to the highly oriented growth along [110] direction. Furthermore, inhibition of bulk and surface charge recombination in NWs results in a larger availability of holes than NRs, thus leading to enhanced photocurrent. Among the samples heated under low temperatures, hematite NCs show an incredible photocurrent thanks to their high carrier density. However, the compact arrangement of NCs leads to a decrease in the number of charge carriers by photogeneration and therefore a limited light absorption. Due to the dense surface, light absorption occurs only on the surface, with a much smaller light contacting area than NWs and NRs, which are more loosely arranged. Second, a higher onset potential in the polarization curve is due to the anodically shifted surface states. Anyway, the highest photocurrent value is recorded for hematite NCs at bias above 1.3 V versus RHE.^[^ ^113^ ^]^

**Figure 3:**
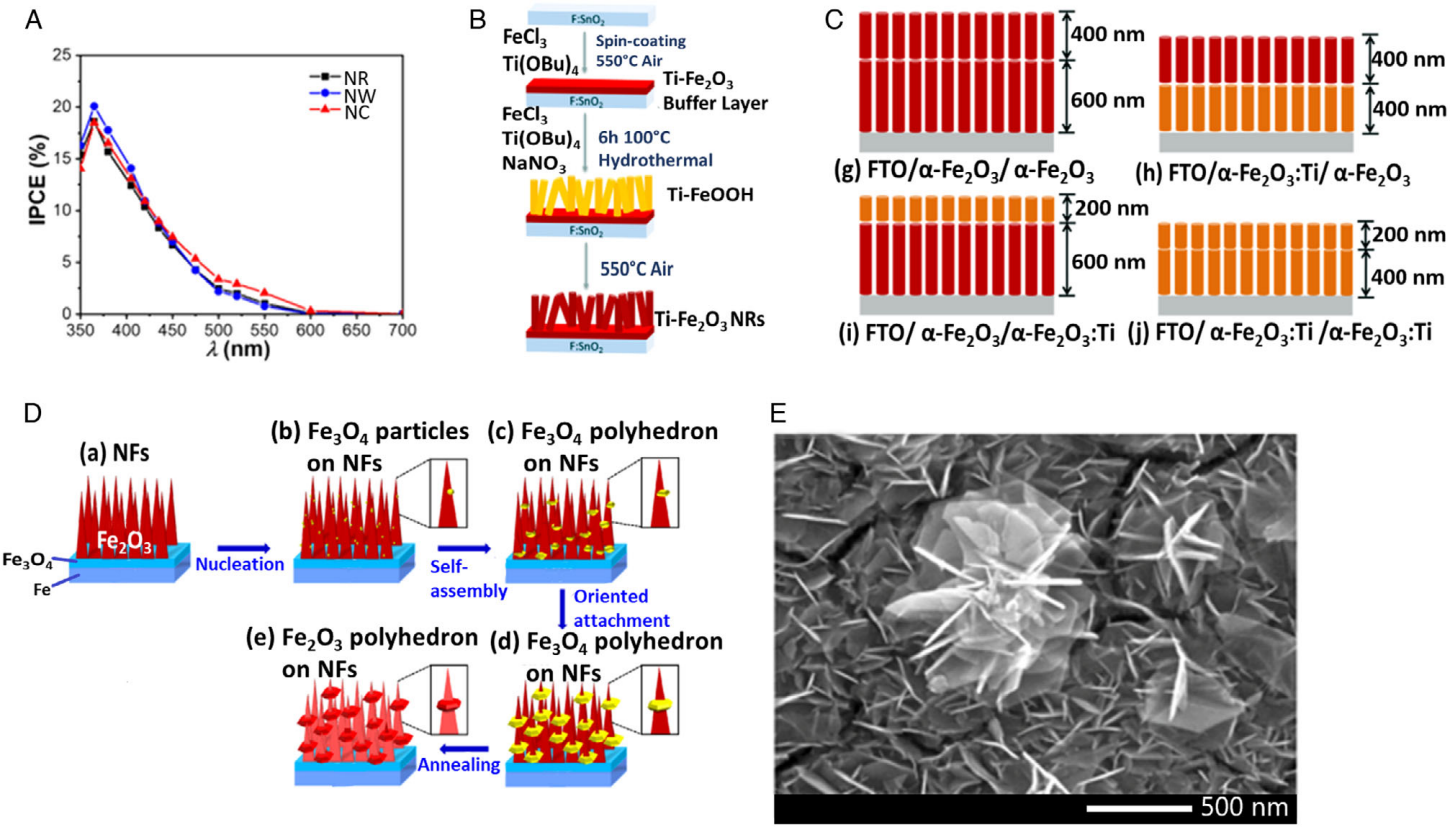
A) IPCE plots of the hematite photoanodes. Reproduced with permission.^[^ ^113^ ^]^ Copyright 2020, Elsevier; B) synthetic steps for the synthesis of Ti‐doped hematite photoanodes. Reproduced with permission.^[^ ^115^ ^]^ Copyright 2019, Elsevier; C) schematic representation of (g) FTO/α‐Fe_2_O_3_/α‐Fe_2_O_3_, (h) FTO/α‐Fe_2_O_3_:Ti/α‐Fe_2_O_3_, (i) FTO/α‐Fe_2_O_3_/α‐Fe_2_O_3_:Ti, (j) FTO/α‐Fe_2_O_3_:Ti/α‐Fe_2_O_3_:Ti. Reproduced with permission.^[^ ^114^ ^]^ Copyright 2019, Elsevier; D) schematic illustration of synthesis process of dodecahedron Fe_2_O_3_ on NFs. Reproduced with permission.^[^ ^126^ ^]^ Copyright 2018, Elsevier; E) FESEM image of iron oxide nanoflowers prepared at room temperature via anodization technique. Reproduced with permission.^[^ ^127^ ^]^ Copyright 2020, Elsevier.

Incident photon‐to‐electron conversion efficiency (IPCE) at 1.23 V versus RHE of the electrodes was measured and estimated, as plotted in **Figure** **3**A. It can be noticed that the photoanodes achieved detectable photoconversion under light wavelength below 600 nm. In particular, NWs show higher quantum efficiency at the short wavelengths, while NCs display an increase in quantum efficiency at long wavelengths, as compared with NR. Orientation plays a significant role in photocurrent efficiency as well. In fact, vertically aligned NRs exhibit a high IPCE (11% and 5% at 360 nm for substrate–electrode (SE) and electrode–electrolyte (EE) illumination, therefore illuminated through substrate/electrode and electrode/electrolyte interfaces, respectively) compared with NPs (1.7% and 0.4% at 360 nm for SE and EE illumination). This is due to the lack of significant grain boundaries in 1D nanostructures and the oriented electron transport toward the back contact. Furthermore, NRs aligned horizontally to the substrate have IPCE around 3% at 360 nm, demonstrating that the orientation with respect to the substrate plays a significant role as well.^[^ ^76^ ^]^

#### 4.2.1 1D Nanostructures: NRs and NWs

As mentioned, NRs are among the most studied nanostructures for the improvement of the PEC performance. While the ways to properly synthesize efficient NRs for WS have been extensively studied in past years, the aim of the latest researches is to improve the efficiency of these nanostructures by exploiting a series of strategies, for instance, introducing a dopant.

Impurity doping has proved to be a facile and efficient way to increase electron density for efficient charge transport, thus improving PEC WS. In particular, α‐Fe_2_O_3_ doped with Ti and Si is the most studied photoanodes. Such approaches are based on Ti‐doping because titanium is completely soluble in the α‐Fe_2_O_3_ structure thanks to the Fe_2–*x*_Ti_*x*_O_3_ solid solution. Doping α‐Fe_2_O_3_ with Ti (up to 20–30 at%) has proved to enhance both the conductivity and the PEC performance.^[^ ^71^ ^]^ This improvement can be attributed to different factors such as the variation of morphology, the increased electron concentration, the suppression of surface state recombination paths due to the Ti^4+^‐induced surface electric field, and the formation of mixed oxide phases that form an overlayer improving the charge separation and transfer at the semiconductor–electrolyte interface.^[^ ^114^ ^]^

Doping of the growth solution (1 or 5 at%) leads to a less oriented deposition of smaller, elongated particles, causing the loss of the ordered quasivertical structure typical of the undoped solution (Figure 3B). In particular, at 1 at% it is still possible to observe the formation of NRs, although with reduced length, while higher doping concentration (5 at%) results in smaller, randomly shaped NPs. The thickness of the film was estimated between 250 and 350 nm, with a slight increase in the case of the 5 at% doped sample; however, this estimate is not completely reliable, given the inhomogeneity of the sample. The decrease in the degree of control on the formation of elongated crystallites toward more randomly oriented spherical particles is confirmed by the XRD patterns, which report a preferential growth along the (110) plane for the undoped sample, while smaller values of the peak ratio (110)/(104) are obtained for 1 and 5 at% doping. Doping with Ti increases the photocatalytic performance of hematite and allows to achieve high photocurrents, with an enhancement about 20% and 30% with respect to undoped hematite, respectively. The reason behind this increased efficiency can be identified as a combination of modified morphology, increased hole diffusion length, and activation of the surface layer. In fact, doping with Ti allows to activate the so‐called “dead layer” by producing a larger number of surface states to trap holes, thus increasing the charge transfer rate and reducing the surface recombination.^[^ ^115^ ^]^

Improvements in the photocurrent density are confirmed by further studies. For instance, Ti‐doping into the bottom and/or top layers of the double‐layered α‐Fe_2_O_3_ NR arrays on FTO reports a good ability to keep the NRs growing along the (110) crystal plane of hematite with a decrease in the film thickness (Figure 3C). Furthermore, the highest PEC performance for WS is obtained with Ti selectively doped into the bottom layer (1.69 mA cm^−2^ at 1.9 V vs RHE under AM1.5G illumination, 4.3 times the undoped FTO/α‐Fe_2_O_3_/α‐Fe_2_O_3_ NR film (0.39 mA cm^−2^).^[^ ^114^ ^]^

Unlike Ti, silicon is not very soluble in iron oxides at ambient pressure and room temperature and PEC performance strongly depends on sample preparation. In particular, the best performing α‐Fe_2_O_3_ films with Si‐doping (i.e., Si 1–2 at%) are obtained through atmospheric pressure chemical vapor deposition (APCVD). Si‐doping in hydrothermally grown α‐Fe_2_O_3_ nanostructures affects their growth and templates the morphological transition from hollow NPs to NWs at 1 at%, while at 5 at% transition from nanostructures with ellipsoidal shape to NWs with preferential growth along the [110] direction and self‐ assembly in layered superstructures is observed. However, only Si‐doping at 1 at% actually improved PEC activity showing a 20% photocurrent enhancement with respect to pure α‐Fe_2_O_3_. On the contrary, the sample with Si content higher than 5 at% showed lower PEC activity due to the lower electrode conductivity caused by increasing Si surface enrichment.^[^ ^71^ ^]^

Further examples of suitable dopants are Sn and Co. The morphology seems to be only slightly affected by the introduction of cobalt: worm‐like shaped NRs are observed both in the case of pristine and Co‐doped hematite with diameters about 100 nm. On the contrary, diameters are slightly reduced for Sn‐doped and (Co, Sn)‐Fe_2_O_3_ NRs. In fact, according to previous studies, the motion of the crystal interface appears to be hindered by the Sn dopant during crystal growth, thus inhibiting the NRs from fusing into the macrostructures. Sn introduced in hematite films results in high photocurrent density, corresponding to 60% enhancement, due to the electron donor activity of the dopant, and thus outperforming the other dopant elements. Co‐doping with other elements such as Co can further improve the photocurrent density as it leads to an improvement in the PEC photocurrent by about 3.0 folds. This is due to the decrease in the charge transport resistance caused by the improved electron and hole separation.^[^ ^116^ ^]^

NWs have been suggested as potential candidates to improve PEC performance. Thanks to their large surface area, and therefore high aspect ratio, they reduce recombination losses and provide a more direct pathway for charge transportation up to the charge collector. Several methods have been investigated for the synthesis of high‐density hematite NWs on conductive substrates. In particular, synthesis of NWs through hydrothermal method has proved to be the most effective to increase the photocurrent density.^[^ ^117^ ^]^ In fact, the 12.9% PCE goal was lowered for hematite NWs, which were previously grown by a hydrothermal method and reached a photocurrent of ≈6 mA cm^−2^. Furthermore, the hydrothermal method is one of the simplest, low cost, and scalable growth approaches.^[^ ^73^ ^]^

Like in the case of NRs, the use of dopants can highly improve the electrical conductivity of hematite because they increase the donor density. One of the most used dopants for NWs is Sn because Sn‐doped α‐Fe_2_O_3_ NWs have shown promising PEC performance. In particular, Sn‐doped hematite leads to a structural distortion of the lattice and therefore exhibits a twofold increase in optical absorption coefficient. Doping can be performed by diffusion from FTO substrate via a high‐temperature annealing process.^[^ ^118^ ^]^

Comparing the annealing with a single‐ or a two‐step approach, higher photocurrents were obtained with the double step (≈0.9 vs ≈0.7 mA cm^−2^). This can be explained by the improvement of structural crystalline order and the increase of dopant in the lattice. In fact, hematite NWs display a preferential crystal orientation along the (110) plane, which confers improved conductivity and improved photocurrents. Increasing the annealing temperature, reduction of the film thickness was observed (from (330 ± 50) nm to (285 ± 37) nm for 550 and 800 °C, respectively), which is consistent with the fact that reduced thickness facilitates the electron pathway toward the charge collector.^[^ ^73^ ^]^

Also, multilayer structures of hematite NWs have attracted a lot of attention.^[^ ^119^ ^]^ In fact, forming a heterojunction interface allows to tune the electronic structure of hematite, reducing the charge recombination at the interface. With this aim, Fe_2_O_3_, ITO/Fe_2_O_3_, ITO/Fe_2_O_3_/Fe_2_TiO_5_, and ITO/Fe_2_O_3_/Fe_2_TiO_5_/FeNiOOH photoanodes were prepared. A quaternary multilayer photoanode with an ITO layer, which is a highly conductive underlayer and can protect against Sn loss from the FTO substrate, Fe_2_O_3_/Fe_2_TiO_5_, an advantageous heterojunction, and FeNiOOH, an efficient oxygen evolution catalysis (OEC) layer, is therefore designed with the aim of reducing the interfacial, bulk, and surface charge recombination and improving its PEC performance. The Fe_2_O_3_ NWs grow vertically aligned on the ITO‐coated FTO substrate with a diameter between 50 and 100 nm. No change in the NW configuration can be noticed upon coating with Fe_2_TiO_5_ onto ITO/Fe_2_O_3_. Furthermore, the ITO/Fe_2_O_3_/Fe_2_TiO_5_ NWs appear to be straighter than the ITO/Fe_2_O_3_ NWs probably due to the effect of confinement by the ultrathin Fe_2_TiO_5_ layer on the layers beneath. The formation of the heterojunction prevents the charge accumulation at the NWs and at the interface, therefore reduces the grain boundary recombination and enhances the photocurrent response. Further photoelectrodeposition with FeNiOOH onto the ITO/Fe_2_O_3_/Fe_2_TiO_5_ NWs leads to no visible nanostructure on the NW surface, evidence of its ultrathin configuration. The current density increases with the following order: Fe_2_O_3_, ITO/Fe_2_O_3_, ITO/Fe_2_O_3_/Fe_2_TiO_5_, and ITO/Fe_2_O_3_/Fe_2_TiO_5_/FeNiOOH, which reaches a large photocurrent of 2.2 mA cm^−2^.

At 350 nm the following IPCE values were obtained: 2.1%, 14.8%, 18.6%, and 28.7% for Fe_2_O_3_, ITO/Fe_2_O_3_, ITO/Fe_2_O_3_/Fe_2_TiO_5_, and ITO/Fe_2_O_3_/Fe_2_TiO_5_/FeNiOOH photoanodes, respectively. The integrated coupling effect of ITO, Fe_2_TiO_5_ and FeNiOOH to the PEC performance is further supported by the large enhancement of IPCE for the ITO/Fe_2_O_3_/Fe_2_TiO_5_/FeNiOOH NWs.^[^ ^120^ ^]^

#### 4.2.2 2D Nanostructures: Films and Nanoflakes

Another successful approach to reduce bulk charge recombination is the synthesis of hematite thin films. As for 1D nanostructures, doping elements can be used to improve the efficiency of the WS process. For instance, Ti‐doped hematite films have been synthesized with a variety of methods including, but not limited to, hydrothermal method, pulsed laser deposition, and dip coating. The as‐synthesized films show morphology with decreased feature size, and therefore increased effective surface area, with respect to the pure films. This results in an increased current density: 0.72 mA cm^−2^ at 1.23 V versus RHE upon doping with 1 at% Ti, compared with 0.30 mA cm^−2^ for pure films.^[^ ^121^ ^]^ It is possible to reduce the surface charge recombination that occurs at the substrate/hematite interface and at the hematite/electrolyte interface by adding an underlayer in the first case, and by using an overlayer or a cocatalyst in the other case. Recently, reduced graphene oxide (RGO) has proved to be an effective solid electron mediator for photocatalytic WS. This can be explained by considering that graphene has a unique planar structure with excellent transparency, large specific surface area, and superior electron conductivity. Therefore, ultrathin hematite films coated with a layer of RGO were synthesized through a separated two‐phase interface hydrolysis method and tested for PEC WS applications under visible light. The photocurrent depends on the film thickness and is improved by increasing the number of deposition cycles. In particular, the maximum photocurrent density is reported for the hematite film with seven deposition cycles (0.61 mA cm^−2^ with an IPCE of 10.4% at 1.5 V vs RHE). The RGO coating on the hematite film further enhanced the photocurrent from ≈0.61 to ≈0.64 mA cm^−2^, corresponding to a maximum IPCE of 18.6% at 400 nm at 1.5 V versus RHE.^[^ ^122^ ^]^ Also mixed α‐Fe_2_O_3_/Fe_3_O_4_ heterostructures generated via CVD of [Fe(OtBu)_3_]_2_ and subsequent thermal oxidation can improve the water oxidation performance with respect to pristine hematite samples. By varying the deposition time, it is possible to tune the film thickness: increasing the growth time from 5 to 45 min, the thickness of the film deposited on the FTO substrate increases from 1.5 μm with smoother surface to 11 μm with micrometer‐sized grains, while that of the top hematite layer increases from 100 to 350 nm. Both films display a top surface covered by irregular grains. Postannealing of magnetite films with thickness higher than 5.6 μm does not lead to the expected oxidation to α‐Fe_2_O_3_ but results in α‐Fe_2_O_3_/Fe_3_O_4_ nanocomposite films with a higher magnetite content toward the bottom of the layer. As this gradient composition/phase allows electron diffusion toward the backside of the electrode through faster iron redox cycling, it supports the carrier separation and transport. These factors lead to an enhancement of the photocurrent density up to 0.48 mA cm^−2^ at 1.23 V (vs RHE), despite a film thickness of 11 μm.^[^ ^123^ ^]^

A significant enhancement of the photocurrent response can be observed also in the case of ultrathin hematite nanoflakes. They are prepared with a low‐temperature fabrication technique with a plasma posttreatment to control the density of oxygen vacancies. Thus, the resulting 2D nanostructures are excellent to decouple the directions of light absorption and photogenerated carrier collection. The plasma treatment is used to activate the surface of the nanoflakes while preserving the nanoflake morphology. The obtained nanoflakes are characterized by thickness of about 20–30 nm and length of 1–2 μm, with preferred orientation along (110) plane and oxygen vacancies. A very high photocurrent density (2.03 mA cm^−2^ at 1.23 V vs RHE) is achieved, which is 12 times higher than that of the as‐prepared hematite without plasma treatment. The highest IPCE was 35.4% at *λ* = 350 nm under 1.6 V versus RHE. This is due to the increase in the density of oxygen vacancies, which decrease the adsorption energy of H_2_O and improve the carrier density. Furthermore, the 2D nanoflake structures with ultrathin thickness also may decrease carrier recombination during the water oxidation process because they help to shorten minority carrier transport length.^[^ ^124^ ^]^

#### 4.2.3 3D Nanostructures: NCs and Nanoflowers

As mentioned, also 3D nanostructures such as NCs are under study because of their ability to improve the PEC performance. As for the other morphologies discussed above, it is possible to further increase the efficiency by taking advantage of other, already mentioned, strategies.

For instance, Ti‐doped hematite film with NC structure can be synthesized by NaF‐assisted hydrothermal deposition on FTO substrate, followed by annealing. Close‐packed NR bundles can be observed on the Ti‐Fe_2_O_3_ film, vertically aligned to the FTO substrate and with preferential orientation along the (110) plane. The subsequent addition of NaF changes the morphology, leading to the formation of the NC structure. Furthermore, the Ti‐Fe_2_O_3_‐NaF film has a highly porous surface structure: this is likely to contribute to the PEC activity because higher reaction rates result from the higher number of active sites caused by a greater surface area. The obtained hematite film shows a high photocurrent density of 2.35 mA cm^−2^ at 1.23 V versus RHE, which is ≈2 times higher than that of Ti‐Fe_2_O_3_ without NaF (1.25 mA cm^−2^). The Ti‐Fe_2_O_3_‐NaF film also displays a higher IPCE value than the Ti‐Fe_2_O_3_ film. In fact, Ti‐Fe_2_O_3_‐NaF at 340 nm reaches an IPCE value of 25.2%.^[^ ^125^ ^]^

α‐Fe_2_O_3_ polyhedrons have proved to be highly promising structures for enhanced PEC performance. In particular, dodecahedral and octodecahedral α‐Fe_2_O_3_ particles produced by a hydrothermal method in the presence of iron chloride and F^−^ anions exhibited improved PEC and photocatalytic performances. This beneficial effect is due to the improved activity of facets of the surface structure, specifically (104) planes, making charge transfer easier. However, the PEC performance is still limited by the low specific surface area of the single crystals. An interesting route to achieve activity and transport improvement of the photoanodes is to combine α‐Fe_2_O_3_ polyhedrons and 1D structures, which can provide directional charge separation and reduced recombination. For instance, α‐Fe_2_O_3_ polyhedrons grown on α‐Fe_2_O_3_ nanoflakes as seeds on a metallic Fe substrate provide an excellent photoanode (Figure 3D). These initial single crystals consist of Fe_3_O_4_ that can be then converted to hematite with high‐index {112} planes in a thermal step. Pristine hematite photoanode reached a photocurrent density of approximately 0.24 mA cm^−2^ at 1.23 V_RHE_, while the dodecahedral α‐Fe_2_O_3_ on NFs photocurrent density is more than 2 times that of pristine hematite (0.57 mA cm^−2^ at 1.23 V_RHE_). The performance can be further improved by doping with Sn. In fact, after Sn doping, the photocurrent density is remarkably enhanced to 2.4 mA cm^−2^ at 1.23 V_RHE_. Next, a FeOOH cocatalyst was decorated on the surface of the α‐Fe_2_O_3_ polyhedron, thus forming crystallized iron oxide during the reaction. This step further enhances the photocurrent density (2.9 mA cm^−2^ at 1.23 V_RHE_) because it increases the charge transfer rate and therefore facilitates the hole transfer at the semiconductor–electrolyte interface. This last photoanode displays the highest IPCE value, with a maximum around 340 nm reaching 66%.^[^ ^126^ ^]^

Another interesting morphology under study is 3D nanoflower‐like structures due to their unique geometry and greater surface area (Figure 3E). These particular nanostructures can be prepared on a large scale through an electrochemical anodization technique at room temperature and are formed by a few dozen nanopetals connected to each other through the center. In particular, the average petal length is about ≈65 nm, while the average thickness is ≈7.7 nm. The photocurrent density shown by the resulting iron oxide nanoflowers upon periodic exposure of UV–vis light is about ≈0.35 mA cm^−2^.^[^ ^127^ ^]^

### 4.3 Role of the Ion Doping over ZnO and α‐Fe_2_O_3_

As mentioned in paragraph 4, hematite and zinc oxide are very promising materials for PEC WS but, unfortunately, their performances are limited by several factors. Doping has proved to be an efficient strategy that can affect the morphology and the catalytic performance.^[^ ^128^ ^]^ Several examples have already been reported in previous paragraphs, so the aim of this article is to describe more deeply the contribution of doping and of different ion dopants.

#### 4.3.1 Doping on ZnO

Doping is one of the main approaches to modulate optical and/or electrical properties of ZnO nanostructures. Metal and nonmetal ions can be easily incorporated into ZnO nanostructures and the introduction of such impurities can be useful to tune the bandgap and the density of the charge carries. Incorporation of ions in the structure affects the electronic structure of the material that subsequently influences the interactions at the ZnO/electrolyte interface. Photoelectrodes based on ZnO are limited in their use due to the wide bandgap of the material itself that impairs the harvesting of light in the visible region. This is the reason why doping is used in the production of highly performant ZnO‐based photoelectrodes.

In the literature, it is reported the synthesis of phosphorous‐doped ZnO NRs deposited on the FTO substrate using CVD method at low temperature. The insertion of phosphorous ions was performed using P_2_O_5_ as a precursor in different ratios and the PEC properties were studied through linear sweep voltammetry using a solar simulator (AM1.5G, 100 mW cm^−2^). The studies were performed in the presence and absence of light irradiation. Under light illumination, photocurrent density values of 0.024, 0.028, 0.035, and 0.031 mA cm^−2^ according to an increasing doping were obtained. The current density is shown to increase when the number of phosphorous ions in the samples increases up to 0.06 g of phosphorous content, while the current density decreases in presence of higher content of phosphorous. The behavior shown by the samples with a low phosphorous content could be ascribed to a higher charge mobility promoted by the phosphorous ions, while the samples with high phosphorous content show a decrease related to changes in the structure. In fact, ZnO changes its structure because of the heavy doping, confirming that the presence of impurities in the structure affects both the performance and the crystallinity of the material. In the reported case, doping improves the overall PEC performance by increasing the charge mobility and by reducing electron–hole recombination. Furthermore, the doped samples show remarkable PCE values of 3.16%, 3.84%, 4.10%, 3.16%, and 1.39%, according to the increasing phosphorous content. It can be concluded that doping with phosphorous increases the electrical conductivity, resulting in a higher transport of charges in the material.^[^ ^129^ ^]^

To further affirm the potentiality of doping in nanostructured ZnO, doping with copper is reported. ZnO NR photoanodes were synthesized through electrochemical synthetic approach. This innovative method allowed to obtain pure ZnO NRs, ZnO:Cu NRs, ZnO:Cu/ZnO NRs, and Cu gradient doped ZnO NRs, whose PEC behaviors were determined through linear sweep voltammetry. In the absence of light, the samples show a negligible photocurrent, while under light irradiation the photocurrent density values increased moderately. The photocurrent densities reached a maximum at a potential of 1.0 V versus saturated calomel electrode (SCE), with values of 83, 69, 52, and 25 μA cm^−2^ for Cu gradient doped ZnO NRs, ZnO:Cu/ZnO NRs, ZnO:Cu NRs, and pure ZnO NRs, respectively. According to the results, the photocurrent response seems to be affected by the presence of Cu ions, and a heavier doping led to a decrease in its value. The hypothesis that could justify this behavior comes from a possible breakdown of the doped phase, producing new centers for the recombination of the charges. Doping with copper ions not only narrows the bandgap of ZnO, enhancing in this way the performances under visible light, but also bends the electronic structure, leading to an increase in charge separation and a reduction of recombination rate.^[^ ^130^ ^]^

Another element involved in the enhancement of ZnO performances is nitrogen. In a study it was demonstrated that the presence of nitrogen impurities in ZnO NW arrays broadens the absorption range with a redshift in the UV–vis region. The presence of such ions did not affect the structure of the material, an effect that often can be observed using the doping approach. The only effect the ions had on the arrays was to decrease the capacitance and increase the photocatalytic efficiency in the visible region. As a consequence, the IPCE under 400 nm wavelength illumination increased from 3.3% to 14.6% after doping. The photocurrent density was measured to be 400 μA cm^−2^ at +1.0 V together with a STH conversion efficiency of 0.15% under a 0.5 V bias. The overall enhancement of the properties of the material comes from the generation of a narrower bandgap and a change in the morphology of ZnO after the incorporation of N, yielding an improved ability of light harvesting in the visible region and a decrease in the resistance. It was demonstrated that N doping can narrow the bandgap of ZnO, but the presence of charged impurities can lower the crystallinity. A recent research mentions that using radiofrequency magnetron sputtering to deposit ZnO‐Al films in N_2_ and O_2_ allows to obtain ZnO thin films codoped with N and Al. They exhibit better PEC performance compared with samples doped with nitrogen alone due to an enhanced crystallinity of the material, demonstrating that the charge‐compensated donor–acceptor codoping strategy is a promising path for engineering the bandgap of many materials and scale up their application in PEC WS.^[^ ^131^ ^]^

#### 4.3.2 Doping on α‐Fe_2_O_3_

Insights in the role of doping have already been given by density functional theory (DFT): for instance, doping with Cu seems to put the VB and CB of α‐Fe_2_O_3_ in a favorable position and decreases the bandgap, so that no external voltage should be needed for WS.^[^ ^132^ ^]^

As already reported, titanium is one of the most noted elements for being able to improve the photocurrent and efficiency. As a further example, Deng et al. reported that the doping of Ti into α‐Fe_2_O_3_ nanostructures changes the morphology from small NR to urchin‐like nanostructures which consist of long NWs on the top of the short NR network on FTO glass. This kind of nanostructures have a larger surface area and smaller feature size, which eventually lead to a high plateau photocurrent density (3.76 mA cm^−2^) and IPCE (60%).^[^ ^133^ ^]^ An IPCE of 62% is reported by Atabaev et al. in the case of 3 at% Ti‐doped α‐Fe_2_O_3_ thin films.^[^ ^134^ ^]^

Apart from the most studied elements, efforts have been dedicated in understanding the role of transition metal dopants. Pan et al. studied the doping of α‐Fe_2_O_3_ by 4d transition metals (Y, Zr, Mo, Tc, Ru, and Rh) first by DFT and then experimentally, demonstrating a high optical absorption and electrical conductivity in the visible part of the solar spectrum for doped hematite. In particular, Ru‐doped films show an almost unchanged morphology, consisting of uniform rod‐like grains. Films doped at 6 and 9 at% exhibit a higher PEC activity than the undoped ones: the photocurrent densities are 0.277 and 0.41 mA cm^−2^ for 6 and 9 at% Ru doping, respectively, while 0.091 mA cm^−2^ for pure at 1.40 V.^[^ ^135^ ^]^

Zr has also attracted attention as a dopant thanks to its ability of improving the charge separation efficiency, through gradient doping, which consists in applying an electric field to the photoanode to bend upward the band, thus helping the electron–hole separation and diffusion in the opposite direction. Chen et al. successfully managed the design and fabrication of α‐Fe_2_O_3_ NR with Zr doping for the *x*‐direction and Sn for the *y*‐direction. SEM images reveal that the morphology is not affected by Sn doping, while a difference can be noticed upon Zn doping: the surface of the NR becomes rough, probably as the result of the formation of branches during the synthetic procedure. The photocurrent of the resulting nanostructure (1.64 mA cm^−2^) appears to be higher than that of both gradient Sn and uniform Zr‐doped α‐Fe_2_O_3_. This enhanced performance with respect to bare α‐Fe_2_O_3_ is due to the increased charge carrier density and conductivity resulting from the incorporation of the two dopants. Furthermore, it is believed that the *x*‐axial gradient Zr doping promoted the charge separation efficiency along the *x*‐direction as the result of an increased bending of the band over a large region of α‐Fe_2_O_3_. Charge separation is further accelerated by the *y*‐axial Sn doping, which lowers the surface trapping states. Therefore, the synergistic effect of these two dopants actually led to an improved PEC WS performance.^[^ ^136^ ^]^

Cu‐doped flower‐like nanostructures were successfully synthesized by Tsege et al. They exhibited a photocurrent approximately fivefold higher (5.34 mA cm^−2^ at ‐0.6 V) with respect to the bare α‐Fe_2_O_3_. This improved PEC performance can be attributed to both the flower‐like morphology and the Cu‐doping because they lead to an enhanced light absorption, thanks to large accessible sites and narrower bandgap, respectively, and charge collection. In particular, the incorporation of Cu improves the charge separation creating p‐conductivity and leads to a shift in the CB, thus making the water reduction reaction more energetically favorable.^[^ ^137^ ^]^

## 5 Junctions

Another option that arose the interest of the scientific community due to unique physical and chemical properties is the formation of heterostructures because they satisfy the requirements for an efficient electrode. Rationally designed heterostructures possess a large interfacial area allowing for rapid charge carrier separation, short diffusion length, improved light absorption, and enhanced charge carrier transport.

In this regard, ZnO‐TiO_2_ core–shell NWs,^[^ ^138^ ^]^ FeVO_4_‐passivated ZnO NRs,^[^ ^139^ ^]^ ZnO/MnO_2_/TiO_2_ NRs,^[^ ^140^ ^]^ Cu_2_O/ZnO p–n junctions,^[^ ^141^ ^]^ ZnO/V_2_O_5_,^[^ ^142^ ^]^ GaON/ZnO,^[^ ^143^ ^]^ and ZnO‐WO_3_^[^ ^144^ ^]^ heterostructures have been reported in the literature to have remarkable PEC performances. Combining properly two different semiconductors (heterostructure formation) may promote the charge carrier separation. Linking together wide and narrow bandgap semiconductors may increase the solar‐driven PEC WS efficiency.

As we already explained before, ZnO and α‐Fe_2_O_3_ cannot be employed in photocatalytic applications without further modification, due to the ZnO low visible absorption, being a wide bandgap semiconductor and the poor charge mobility in α‐Fe_2_O_3_. For this reason, it is of extreme importance to the engineering of these metal oxides semiconductor materials, in order to obtain an efficient solar‐driven photocatalytic hydrogen evolution.

Combining together ZnO and α‐Fe_2_O_3_ materials at the nanoscale, nanocomposites can be formed, bringing to the formation of a Z‐Scheme. In this case, when ZnO and α‐Fe_2_O_3_ are excited under solar illumination, the photogenerated electrons rapidly migrate from CB of ZnO to VB of α‐Fe_2_O_3,_ leaving the holes accumulate in the ZnO VB and electrons in high potential α‐Fe_2_O_3_ CB, available to reduce H^+^ in H_2_. Therefore, there is a decrease in the charge recombination probability and the PEC performance is enhanced. However, the PEC WS efficiency can increase only when several requirements are simultaneously fulfilled: proper energy band alignment is necessary, but it is not enough. Therefore, morphology becomes crucial at this point. Accurate engineering and design of the photocatalyst may overcome problems related to charge carrier transport and recombination. In fact, many evidences prove the existence of a correlation between structures with reduced dimensions and improvement on photocurrent density, associated with the presence of defects in nanostructures.^[^ 100, 110 ^]^

As described in the previous paragraphs, both α‐Fe_2_O_3_ and ZnO have been synthesized in a wide variety of structures and nanoshapes. New photoelectrodes can be obtained, by combining these two materials with different shapes and morphologies, benefitting of both new structural features and electronic properties.

### 5.1 Films and 0D Structures

In view of their unique optical, electronic, and surface properties, it is of great interest to the use of 0D nanostructures, in form of NPs or QDs, as photoactive materials under sunlight,^[^ 145, 146 ^]^ despite the challenge to produce quantum confined wide bandgap semiconductors, which require lateral dimensions as small as few nanometers.

**Figure 4:**
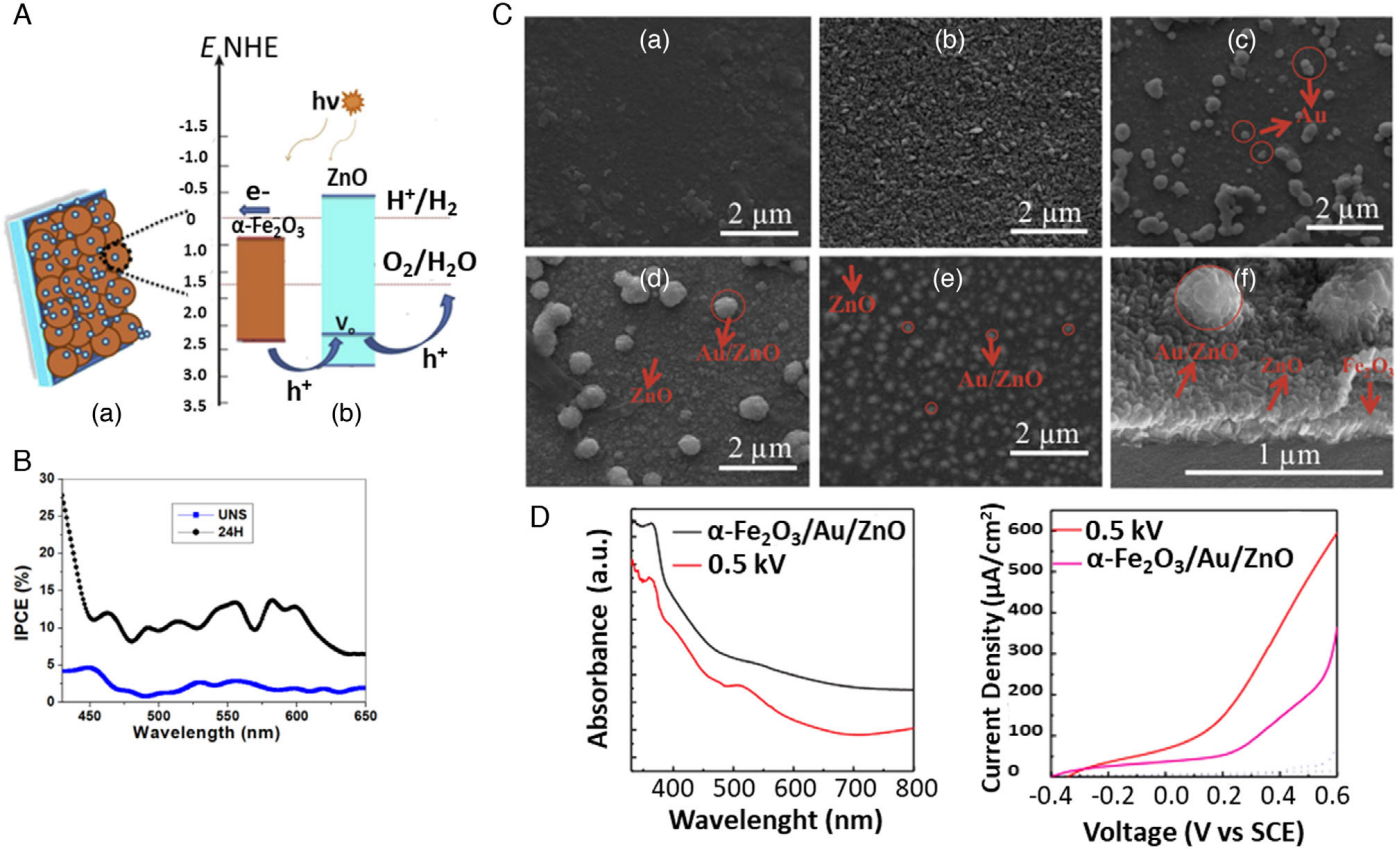
A) Arrangement of ZnO QDs over α‐Fe_2_O_3_ thin film (left) and band edge energetic and photogenerated carrier flow at ZnO QDs over α‐Fe_2_O_3_ film/electrolyte interface (right). B) IPCE spectra for unsensitized (UNS) and 24 h ZnO QDs sensitized α‐Fe_2_O_3_ thin films. Reproduced with permission.^[^ ^147^ ^]^ Copyright 2015, Elsevier. C) FESEM micrographs of (a) α‐Fe_2_O_3_, (b) ZnO, (c) α‐Fe_2_O_3_/Au, (d) α‐Fe_2_O_3_/Au/ZnO, (e) α‐Fe_2_O_3_0.5 kV/Au/ZnO, (f) cross section of α‐Fe_2_O_3_/Au/ZnO. D) On the left, α‐Fe_2_O_3_/Au/ZnO and α‐Fe_2_O_3_0.5 kV/Au/ZnO samples. On the right, α‐Fe_2_O_3_/Au/ZnO and α‐Fe_2_O_3_0.5 kV/Au/ZnO samples under irradiation (100 mW cm^−2^) in 0.5 m NaOH. Reproduced with permission.^[^ ^150^ ^]^ Copyright 2018, Elsevier.

In this frame, Satsangi and co‐workers^[^ ^147^ ^]^ investigated the effect of ≈2 nm ZnO QDs sensitization times (24, 48, and 72 h) on α‐Fe_2_O_3_ electrodeposited FTO thin films for PEC (**Figure** **4**A). According to their results, the amount of ZnO QDs deposited (proportional to the sensitization time) is crucial in determining the PEC performance (optimum sensitization time of the study equal to 24 h). The higher the deposition time is, the more QDs aggregation occurs. To better understand the structure and morphology of the surface heterostructure, they measured the average thickness and roughness of these films. An increase in thickness (from 900 to 950 nm) and a decrease in roughness (from 240 to 185 nm) are noticed, indicating that some QDs are deposited inside the unsensitized film pores (smoothening the surface), others on the surface (thickening the film). For what concerns the optical properties, the absorbance decreases in the visible range and increases in the UV region, in presence of ZnO QDs, compared with the bare α‐Fe_2_O_3_ film and this is not due to a widening in the hematite bandgap, which remains fixed at ≈1.9 eV, but to the presence of ZnO QDs over α‐Fe_2_O_3_ thin film, as they are well known to absorb in the UV region. However, they stressed the importance of QDs sizes on the widening of their bandgap. Quantum confinement effect increases the bandgap of QDs and creates discrete energy bands, which is expected to lead a more favorable band energetic for the injection of photogenerated charge carriers. There can be several reasons such as the matching band edges of QDs with the semiconductor for carriers transport, together with hole or electron trapping in a bandgap state, competing with the fast relaxation of carriers to the ground state and finally, the possibility to have long lifetime of lower energy photoexcited state, so that photoinduced tunnelling between the dot and the semiconductor can occur.^[^ ^148^ ^]^ The highest photoresponse in the 24 h sensitized film in comparison to the other samples is due to the lower amount of agglomerated QDs. In fact, the presence of agglomerated QDs is the main reason of the decrease in PEC performances, mainly for three reasons: 1) the pores on α‐Fe_2_O_3_ film are gradually blocked by the agglomerates, causing the redox couple ion transport hindered in the nanoporous network; 2) a large amount of agglomerated QDs leads to an higher surface coverage of the film and, consequently, shields the high energy photons which have shorter penetration depth than that with longer wavelength; and 3) nonactive agglomerated QDs can act as charge recombination centers.^[^ 148, 149 ^]^ The 24 h sensitized‐film shows the best PEC performance according to the higher photocurrent density (*J*_ph_) of 2.84 mA cm^−2^ (at 0.75 V/SCE under 150 mW cm^−2^ illumination intensity) and an increased IPCE value over the scanned wavelengths (Figure 4B). Furthermore, the H_2_ evolution was found to be 6 mL h^−1^ cm^−2^ at 0.75 V/SCE. This is attributed to the better separation and transport of photogenerated charge carriers in the presence of ZnO QDs and a larger depletion layer width combined with higher open‐circuit potential.

In the work of Kant et al., α‐Fe_2_O_3_/Au/ZnO heterostructured thin films were fabricated via chemical spray pyrolysis method, starting from the deposition of α‐Fe_2_O_3_ spherical NPs (100–130 nm) on ITO glass substrate followed by Au NPs (200–300 nm) layer and ZnO thin film covering the whole surface.^[^ ^150^ ^]^ FESEM analysis (Figure 4C) confirmed that a noncontinuous Au NPs layer covered the surface of the underlying hematite coating and ZnO nanograins covered both α‐Fe_2_O_3_ and Au spherical NPs. An additional voltage of 0.5 kV applied during the deposition of α‐Fe_2_O_3_ thin films significantly affected the morphology and subsequently the optoelectronic properties of the whole heterojunction, creating more effective interfaces between Au nanospheres and hematite. Furthermore, Au NPs sandwiched film promoted the charge transport from ZnO to α‐Fe_2_O_3_ CB thanks to a suitable band alignment and surface plasmon resonance (SPR) effect (Figure 4D, left). Enhanced photocurrent density, as high as 500 μA cm^−2^ (at 0.5 V vs SCE) was achieved (Figure 4D, right).

Yoon and co‐workers proposed a cold‐spray‐coated Fe_2_O_3_ photoanode on ITO substrate, passivated with ultrathin ZnO/TiO_2_ overlayers, deposited via atomic layer deposition (ALD).^[^ ^151^ ^]^ From the water contact angle (WCA) measurements, the deposited passivating overlayers significantly affected the surface morphology of Fe_2_O_3_ underlying film. In fact, the roughness of the surface increased, compared to the bare Fe_2_O_3_ film, and the wettability decreased (increasing in WCA), owing to the formation of spherical electrolyte droplets. However, the overlayers protected the underlying film and enhanced PEC performance, by reducing the surface defects and limiting the hole trapping. Water oxidation was promoted thanks to the one‐way hole transfer reaction from the passivation layers to the EE interface, thus suppressing the electron–hole recombination processes. Further effect of these passivating layers on PEC performances was the increase in photogenerated charge carrier lifetime. Moreover, the photocurrent density of bare Fe_2_O_3_ films improved significantly, when the ultrathin ALD‐based ZnO/TiO_2_ coating was applied. In fact, the *J*_ph_ value increased from 0.5 up to 4.25 mA cm^−2^.

Another study is about the incorporation of MoS_2_ NSs into a Fe_2_O_3_/ZnO composite system. The bare material consisting of α‐Fe_2_O_3_/ZnO was studied after 4 h of illumination and produced ≈480 μmol H_2_ g^−1^ catalyst. The generation of hydrogen through WS was further enhanced by using as electrocatalyst a composite material made of α‐Fe_2_O_3_/ZnO incorporated with MoS_2_ NSs. This heterostructure was able to generate ≈610 μmol H_2_ g^−1^ catalyst after 4 h of solar light irradiation. The production rate was larger compared to other materials, such as analogous metal oxides and transition metal dichalcogenide based nanocomposites reported in the literature. The enhancement in the photocatalytic efficiency of α‐Fe_2_O_3_/ZnO after the incorporation of MoS_2_ NSs was attributed to the structure, as well as to the large surface to volume ratio, effective separation, and transport of charges at the interface of the heterojunctions.^[^ ^152^ ^]^

Another case reported is about ZnO particles decorated with hematite NPs. The formation of such heterojunction allows to obtain current density values of 0.77 mA cm^−2^ at 1.2 V in the presence of light. Under dark conditions, bare ZnO shows a current density of 0.16 mA cm^−2^, so consequently it may be observed that the addition of hematite increases the current density of bare ZnO under both the light and dark conditions of ≈0.61 mA cm^−2^. This kind of result demonstrates that the formation of heterojunctions between the two considered materials is a promising approach for enhancing the performances for PEC applications. This is further supported by the analysis of the photoresponsive properties of FTO/ZnO/Fe_2_O_3_ electrodes at 1.2 V versus Ag/AgCl. The characterization of this composite material emphasizes a fast recombination on the surface of the photoelectrodes because of the limited lifetime of the generated e–h^+^ pairs. The FTO/ZnO/Fe_2_O_3_ electrode can reach a current density of 0.70 mA cm^−2^ when exposed to light, a value that is 1.75 times higher than the one obtained under dark conditions.^[^ ^153^ ^]^

Similarly, in the literature it has been reported the case of a ZnO/Fe_2_O_3_ heterojunction synthesized by sequential depositions of α‐Fe_2_O_3_ and ZnO films on FTO. Chronoamperometry studies were used to determine the photocurrent density for α‐Fe_2_O_3_/FTO and α‐Fe_2_O_3_/ZnO/FTO electrodes. The respective photocurrent density values were 0.4 and 2.4 mA cm^−2^, which was 6 times bigger than the first mentioned electrode. The increase in the photocurrent density comes from the synergic effect of two factors: the generation of an electric field in the heterojunction, which suppresses the recombination of the photogenerated charge carriers, and the application of an appropriate external bias, which favors the separation and transfer of the charge carriers. Moreover, the overall enhancement in the photocurrent density can be also attributed to the improved light harvesting ability of the material itself, coming from the adequate band‐edge alignment of the two semiconductors involved in the formation of the heterostructure.^[^ ^154^ ^]^

Significant gains in PEC WS photocurrent were reported using n/n junction bilayered nanoheterostructured thin films ZnO/Ag‐(α)Fe_2_O_3_ as well. The heterostructures were able to yield from 2‐ to 20‐fold increment in photocurrent. During the characterizations, a photocurrent under anodic bias appeared, confirming the n‐type nature of the thin films. Moreover, the introduction of Ag in α‐Fe_2_O_3_ overlayer further increased the photocurrent density compared to monolayered pristine α‐Fe_2_O_3_ films. It must be pointed out that many works report the detrimental effect that high concentration of impurity's incorporation has on the PEC response. In fact, heavy concentrations of impurities can act as recombination sites. This is the reason why in this case the optimum amount of Ag in the material was found to be 3 at%. This is evidenced by the presence of sharp declines in the photocurrent density of samples containing higher amounts of Ag. The overall enhancement in PEC WS can be ascribed to the high surface area and to the formation of an n/n heterojunction, responsible for an efficient separation and transfer of the charges.^[^ ^155^ ^]^

#### 5.1.1 NR/NW Arrays

**Figure 5:**
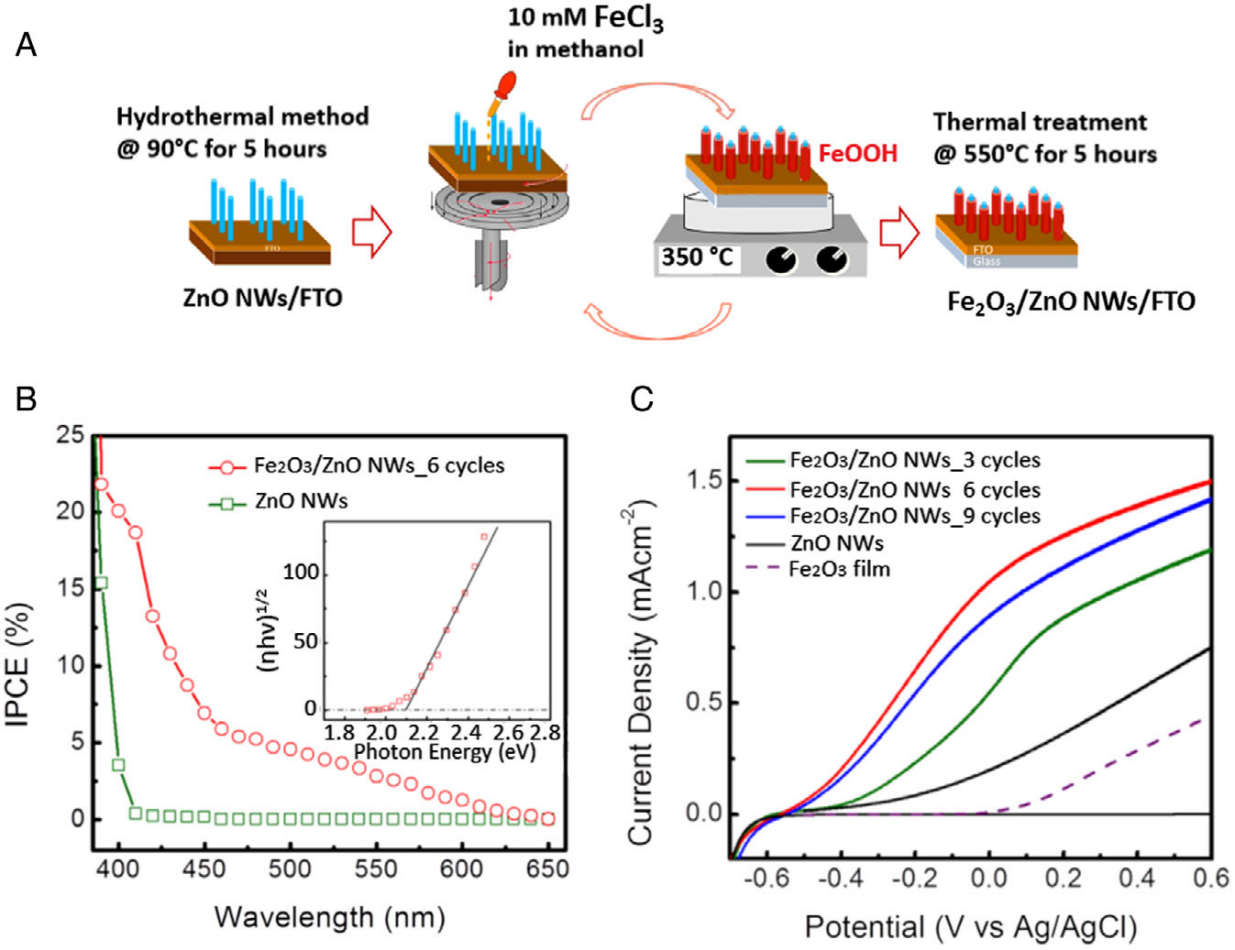
A) Scheme of the synthesis of ZnO/Fe_2_O_3_ core–shell NW array. B) IPCE as a function of excitation wavelength at a potential of −0.2 V from ZnO/Fe_2_O_3_ core–shell NWs at three cycles of deposition annealing procedure; inset, variation of the square root of IPCE (h) times hν with photon energy for ZnO/Fe_2_O_3_ core–shell NWs. C) Photocurrent–voltage responses of the ZnO/Fe_2_O_3_ core−shell NWs. Reproduced with permission.^[^ ^156^ ^]^ Copyright 2015, American Chemical Society.

One of the most studied morphologies for ZnO/α‐Fe_2_O_3_ is the core–shell structure. Here, we report a significant example from Lin and co‐workers.^[^ ^156^ ^]^ They proposed an easy two‐step synthesis strategy to obtain ZnO/α‐Fe_2_O_3_ n/n heterojunction: the ZnO NW array hydrothermally growth followed by the wet‐chemical impregnation of FeCl_3_ solution annealed in air at 550 °C (**Figure** **5**A). α‐Fe_2_O_3_ shell and ZnO NWs array were compactly and uniformly grown on the FTO glass surface with a length over 2 μm and diameters between 50 and 80 nm. The morphology after the FeCl_3_ wet‐chemical deposition and annealing gave clear evidence of the formation of α‐Fe_2_O_3_ thin layer covering the ZnO NWs. The roughness and thickness increased, with an increasing number of deposition cycles and annealing processes. However, the overall density and lengths of core–shell structures remained constant; thereby this method did not damage the ZnO NWs. The best performing sample was obtained with six cycles of deposition of the shell layer with enhanced IPCE at −0.2 V and increased photocurrent density of 1.5 mA cm^−2^ at 0.207 V versus RHE (Figure 5B,C). In fact, when the α‐Fe_2_O_3_ thickness increases, the photocurrent density decreases. This can be ascribed to the short lifetime of photogenerated e^−^–h^+^ pairs and the higher bulk charge recombination processes.

Also, ZnO/Fe_2_O_3_ core–shell NWs grown on FTO substrate through a simple wet‐chemical process are reported to be efficient photoelectrocatalysts for WS applications. In fact, after the deposition of a thin layer of α‐Fe_2_O_3_ acting as a shell on the surface of the ZnO NWs, the photocurrent increases rapidly up to 1.05 mA cm^−2^. As a comparison, the photocurrent was demonstrated to be more than double than bare ZnO NW and comparing with each other all the samples, an appreciable photocurrent under visible light irradiation is observed as well. These results can be ascribed to the orientation of the NWs that favors physical matching. This provides a direct conduction pathway for the electrons and impairs the diffusion of photogenerated holes.^[^ ^157^ ^]^

**Figure 6:**
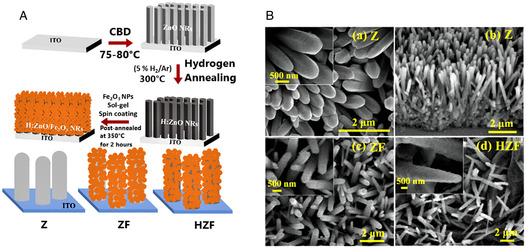
A) Schematic showing fabrication process for H:ZnO/Fe_2_O_3_ NRs and different samples investigated in the study. B) FESEM images of the (a) pure ZnO NRs (Z), (b) cross‐sectional of ZnO NRs, (c) ZnO/Fe_2_O_3_ NR arrays with two deposition cycles of Fe_2_O_3_ NPs (ZF), (d) hydrogenated ZnO/Fe_2_O_3_ NR (HZF) arrays with six cycles of Fe_2_O_3_ NPs. Inset shows the enlarged image of respective samples. Reproduced with permission.^[^ ^158^ ^]^ Copyright 2020, Elsevier.

Mehta and co‐workers present another example on the use of 0D structures^[^ ^158^ ^]^: the growth of randomly oriented heterostructure of hydrogenated ZnO NRs array decorated with Fe_2_O_3_ NPs (H:ZnO/Fe_2_O_3_, HZF) (**Figure** **6**A). Their work was focusing on the decoration of hydrogenated ZnO NRs array on ITO‐coated glass substrate, with Fe_2_O_3_ NPs by a simple sol–gel spin coating process varying the number of deposition cycles (*n*) from 0 to 8 obtaining HZF samples with an increasing thickness (labeled as HZFn samples with *n* from 2 to 8). First, they focused on how the changing in *n* affected the NRs morphology. As shown in FESEM images (Figure 6B), ZnO NRs diameter typically was in the range of 400–500 nm with a favored *c*‐axis growth. Additionally, the hydrogenation did not significantly affect the morphology. After the Fe_2_O_3_ NPs decoration, the NRs morphology changed. For *n* ≥ 4 the surface of ZnO NRs was uniformly covered. Fe_2_O_3_ NPs deposition affects the PEC performances of H:ZnO/Fe_2_O_3_ NR samples. The photocurrent density (*J*_ph_) increased at higher Fe_2_O_3_ NPs deposition cycles, up to HZF6, which indicates an enhanced visible light activity and a more efficient photogenerated charge carrier separation. However, for HZF8 sample, the *J*_ph_ decreased, indicating an increased bulk charge recombination, due to the higher Fe_2_O_3_ thickness. Thus, HZF6 was the optimal sample with a *J*_ph_ of 6.8 mA cm^−2^, an improved visible light absorption and the suppression of photogenerated charge carrier recombination, due to the stair‐like band alignment. Furthermore, the PCE improved for HZF6 with an increment of 1.13% compared to HFZ2.

Thickness is a critical parameter, when dealing with α‐Fe_2_O_3_, due to its reduced charge mobility. Commandeur et al. designed a 3D triple‐layer heterostructure.^[^ ^159^ ^]^ Hematite (α‐Fe_2_O_3_) crystals were electrochemically deposited with an optimized thickness over vertically aligned Y‐doped ZnO (YZnO) NRs with a thin layer of sandwiched ZnFe_2_O_4_, which contributed to the reduction of charge recombination. The TEM images show clearly the formation of a triple‐layer heterostructure; XRD confirmed that the 8 nm‐coated film of ZnFe_2_O_4_ directly attached to YZnO NRs and a mesoporous network of α‐Fe_2_O_3_ NPs, with an average size of 16 nm, covered the whole NR surface. The electrochemical deposition method is important, to better control the morphology of hematite coating. A series of advantages can be recognized, such as the good electrical contact between interfaces (YZnO/ZnFe_2_O_4_ and YZnO/α‐Fe_2_O_3_), enabling an easier charge transfer and the possibility to control the hematite domains. In fact, in the case of iron phase, typically showing a small minority carrier path length, the control on the morphology coating is needed to have small enough iron domains so that charge can reach faster the surface reaction centers before the recombination process occurs. Furthermore, NPs network increased roughness, hence surface area, promoting the hole transfer to electrolyte. The formation of the triple junction had the effect to sensitize the absorption of light from UV to visible range and charge separation was enhanced, thanks to the Fermi levels alignment, which induced the onset of favorable electronic band structure. In fact, the proper position of VB gave the possibility to have rapid hole transfer from YZnO core through ultrathin ZnFe_2_O_4_ film to the surface α‐Fe_2_O_3_ NPs. Similarly, the CB alignment allowed the electrons cascade to ZnO core, which hindered the h^+^–e^−^ recombination, and enabled high rate H_2_ evolution at the electrode surface. This was proved with an intense improvement in PEC WS performances: the photocurrent density was 1.59 mA cm^−2^ at 1.23 V_RHE_ (2.4 times higher than the uncoated ZnO NRs) and IPCE clearly increased in the UV–vis range.

**Figure 7:**
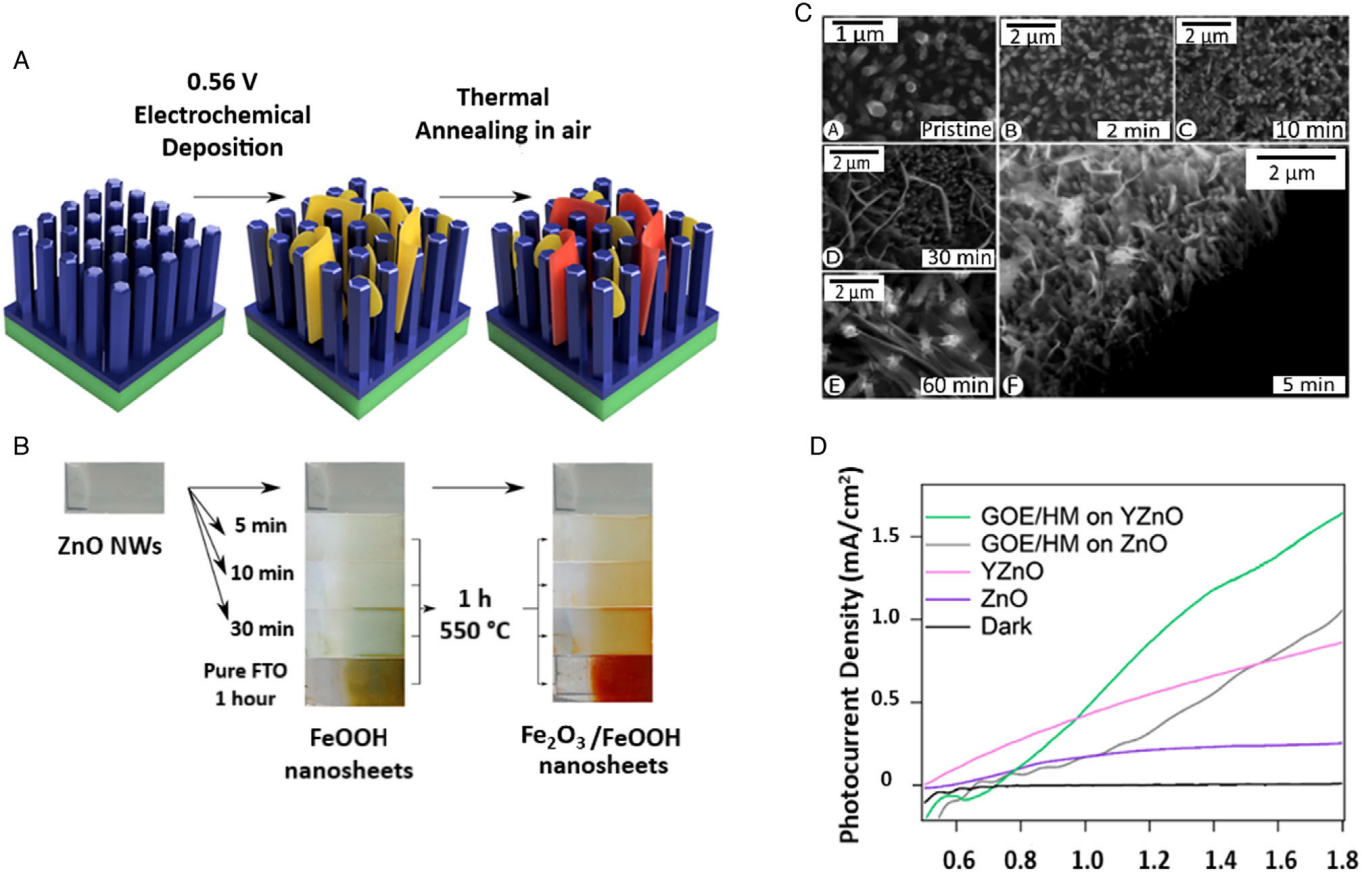
A) Schematic for the deposition process, B) along with sample photographs. On the left, it shows the pristine YZnO NRs, with no coating, and in the center, the NRs with FeOOH NS coating falling between the NRs after electrodeposition. On the right, the conversion of some α‐FeOOH to Fe_2_O_3_ by thermal annealing in air. C) Top‐down SEM images of (A) YZnO NRs and coated samples after annealing; (A–E) the morphology shows increased Fe_2_O_3_ NSs with greater deposition duration. The angled cross‐sectional image of the 5 min deposition sample is shown in (F). D) Comparison of PEC WS performances in ZnO and YZnO with and without 5 min GOE/HM NS coatings. Reproduced with permission.^[^ ^160^ ^]^ Copyright 2021, American Chemical Society.

In a more recent research from the same group, the synthesis of hybrid polycrystalline goethite (α‐FeOOH) and hematite (α‐Fe_2_O_3_) NSs (GOE/HM NSs) on yttrium‐doped ZnO NRs (YZO NRs) is proposed using anodic electrochemical deposition.^[^ ^160^ ^]^ Proper engineering of the morphology is crucial in designing a promising system for PEC WS. In fact, GOE/HM NSs were wrapped around, rather than above, the YZnO NRs (**Figure** **7**) and this unique setup was the key to get higher performances. The electrolyte can diffuse and be in contact directly with NRs. The authors also showed how the density and NSs surface area were of extreme importance; by optimizing these two parameters, a balance between absorption of light and charge transport to the electrolyte was achieved. The highest absorption of visible light in the range of 450–550 nm was achieved with the highest coating times (30 min), but the best PEC performances were obtained with 5 min of deposition at a fixed potential (0.56 V). This means that thinner NSs do not limit the electrolyte and gas transport in and out of the film, provide a good contact with the electrolyte, and decrease hole path length, thus reducing charge recombination. The synergistic effects of hematite/goethite NSs with YZnO NRs resulted in UV–vis absorption and good IPCE, which demonstrated a proper band alignment at interfaces, giving a reduced band gap energy. Overall, the hybrid material showed an enhancement in WS activity, giving a photocurrent density of 0.92 mA cm^−2^ compared to that of pristine ZnO NRs, which was 0.2 mA cm^−2^ (Figure 7D).

As mentioned before, heterostructures retain a remarkable potentiality in PEC applications due to the combined interactions of two different materials. This is the case, for instance, of Fe_2_O_3_ NRs decorated with a thin ZnO layer obtained through hydrothermal method. This approach allowed to reach average photocurrent density values of 0.033 and 0.038 mA cm^−2^, proofing that the construction of a heterogeneous system with ZnO improves the photoelectric conversion efficiency. This decoration with a thin ZnO coverage layer of the surfaces of the Fe_2_O_3_ NR enhanced the light harvesting ability of the overall material and showed an increased charge separation efficiency in the Fe_2_O_3_–ZnO composite system. This is motivated and expected because of the suitable band alignment between the Fe_2_O_3_ and ZnO.^[^ ^161^ ^]^

**Table** **2** summarizes some of the reported emerging photoactive materials based on ZnO and hematite. The purpose of this table is to give an overview on the most promising differently shaped nanomaterials as efficient and low‐cost photoanodes for PEC WS application. Furthermore, we hope to facilitate the reader to compare the performances related to the morphology and the composition of the aforementioned materials.

**Table 2:** Efficient and low‐cost 0D, 1D, 2D, and 3D photoanodes for PEC WS

Material	Morphology	*J*_ph_ [mA cm^−2^]	Ref.
A. ZnO			
1. BiVO_4_/ZnO QDs	0D	5.50 (1.23 V vs RHE)	[84]
2. ZnO NRs/CdS QDs	1D	9.16 (0.4 vs SCE)	[88]
3. ZnO/CdS/CdSe on Zn foil substrate	1D	6.24 (−0.2 V vs Ag/AgCl)	[89]
4. Ge‐doped ZnO NRs	1D	12.0 (1.654 V vs RHE)	[91]
5. ZnO NRs/NSs – CdS QDs	2D	4.65 (0.4 V vs Ag/AgCl)	[106]
6. a‐nanosails	2D	0.62 (0.2 V bias)	[109]
7. Star‐like ZnO	3D	0.31 (1.23 V vs RHE)	[110]
8. Flower‐like ZnO	3D	0.34 (1.23 V vs RHE)	[110]
B. α‐Fe_2_O_3_			
1. Ti‐doped FTO/ α‐Fe_2_O_3_/ α‐Fe_2_O_3_ NRs	1D	1.69 (1.9 V vs RHE)	[114]
2. α‐Fe_2_O_3_ NR arrays	1D	≈ 6.0 (1.23 V vs RHE)	[73]
3. ITO/ α‐Fe_2_O_3_ /Fe_2_TiO_5_/FeNiOOH NWs	1D	2.20 (1.23 V vs RHE)	[120]
4. Ti‐doped α‐Fe_2_O_3_ films	2D	0.72 (1.23 V vs RHE)	[121]
5. RGO coating α‐Fe_2_O_3_ ultrathin films	2D	0.61 (1.50 V vs RHE)	[122]
6. Ultrathin α‐Fe_2_O_3_ nanoflakes	2D	2.03 (1.23 V vs RHE)	[124]
7. α‐Fe_2_O_3_ polyhedron on nanoflakes	3D	2.90 (1.23 V vs RHE)	[126]
C. Composites			
1. ZnO QDs sensitized α‐Fe_2_O_3_ thin films	0D	2.84 (0.75 V vs SCE)	[147]
2. Ultrathin ALD‐based ZnO TiO_2_ coating on α‐Fe_2_O_3_ films	0D	4.25 (0.7 V vs Ag/AgCl)	[151]
3. FTO/ZnO/ α‐Fe_2_O_3_	0D	0.70 (1.2 V vs Ag/AgCl)	[153]
4. ZnO @ α‐Fe_2_O_3_ NW arrays	1D	1.5 (0.207 V vs RHE)	[156]
5. H:ZnO/ α‐Fe_2_O_3_ NRs	1D	6.8 (1.23 V vs RHE)	[158]
6. YZnO/α‐Fe_2_O_3_	1D	1.59 (1.23 V vs RHE)	[159]

## 6 Conclusions

This review focused on an interesting aspect: how morphology affects PEC WS in metal oxide photocatalysts based on ZnO, α‐Fe_2_O_3,_ and their composites. We targeted the investigation on these two specific materials and their composites because they represent, to our view, among the most promising materials in the field. Nowadays, one of the most relevant problems of such a field is that materials currently studied have limited performances, exhibit low efficiency, and are not eco‐friendly. All these features make them unsuitable for commercialization. Bare materials themselves offer limited chances to succeed. For this reason, we considered the possibility to reach highly complex nanostructures, starting from easily accessible materials such as ZnO and Fe_2_O_3_. The first target was to highlight the improvements in the efficiency brought from different morphologies and the possibility to combine them, reaching promising heterostructures. The second target was to identify the physical and chemical processes behind this increase in functionality. Recent advancements were made to obtain peculiar nanoshapes: from 0D to 3D structures. Several examples of these materials were reported to improve in the overall performances thanks to their structure. Starting from common shapes used as building blocks, complex structures have been synthesized. The effort to obtain new complex shapes arises from the fact that simple nanostructures are not sufficient themselves to boost H_2_ evolution, while few recent studies reported combinations that showed new properties coming from the synergistic effect of different morphologies.

The materials that have been exploited showed remarkable improvements in the ability to generate an adequate photocurrent density. Successful strategies involve application of 0D, 1D, 2D, and 3D structures. 0D materials have an enormous potential that we can take advantage of because their incorporation broadens the light absorption range especially toward visible region and suppresses charge recombination. However, they show a nonnegligible problem related to their partial aggregation that negatively influences their functionality, due to the decrease in specific surface area and the thickening of the layer covering other nanostructures. This represents a challenge, to open up to new prospects and implementations in this field, having isolated single layers of 0D structures deposited on top of other nanostructures. 1D materials have been extensively studied in almost all the possible shapes that include in some cases quantum confinement in one of the three dimensions. Their properties in applications such as PEC WS are well known. 1D structures alone are sufficiently performant but some limits, such as altered thermal conductivities and lowered melting points, can impair their stability and efficiency. Their coupling with other nanostructures enhances even more some of the most important parameters in PEC WS, as the ability to harvest light, separate charges, increase the specific surface area and the stability of the materials themselves. 3D structures have been proposed as well, with multiple shapes and variable performances, further confirming that a rational design and assembly of standard building blocks can give an important contribution in boosting PEC WS efficiency.

The present technology offers consolidated techniques to synthesize different morphologies for ZnO and Fe_2_O_3_. Capitalizing on such advancement in synthetic routes, we focused on the role of morphology, sometimes underestimated or not completely exploited in its full potential.

A great issue affecting in a broader view the full subject of PEC WS, including the materials described here, is the challenging comparison of the results obtained from different studies, due to the lack of a standard protocol to follow for the efficiency measurements. As stated by Gogotsi et al.,^[^ ^162^ ^]^ the absence of a standard reference makes the understanding and critical discussion of the results complicated. In addition, results are often not reported in a suited way, not allowing the reader to immediately catch the meaning of the research and to obtain a fair comparison of different studies.

For future work, further modification and engineering of the nanostructures may offer interesting advantages, in terms of improvement of the PEC performance. The introduction of flexible substrates as well as the introduction of solvents into the synthesis protocols admit the production of better‐defined nanostructures, being more flexible and homogenous. Such characteristics are some of the most sought‐after ones because they could promote the scaling up of the production and hence expand the range of application. Composite nanostructures offer several remarkable properties that otherwise would not be fully exploited in the case of bare materials. Sensitizing with one or more materials opens the way to the tuning of the properties, extending the absorption spectrum, engineering the bandgap, increasing the surface‐to‐volume ratio, increasing the resistance toward corrosion, and so on. We reported here several different morphologies with interesting efficiencies, alongside with the more studied ones such as 1D nanostructures. Therefore, trying to further modify them, for instance, by decreasing the dimension of NCs, or combining different types, such as combining 1D morphologies with higher order ones, appears as a promising approach. In particular, exploring more in depth the fabrication of multimaterials systems would allow empowering the overall photocatalyst because each component, main catalyst, cocatalyst, doping elements, gives a specific contribution in the modulation of the light management and transport of the photogenerated charges. In fact, being able to reduce the charge losses during transport and collection of the photogenerated charges is still one of the main challenges. This could be applied with particular focus to the materials discussed in the review, thus ZnO, Fe_2_O_3_, and their composites, because they showed promising properties, which could be employed not only in academic research but also in more practical applications. Further deepening the knowledge about particular morphologies, optimization of the different types reported and fabrication of composite systems are all approaches that could allow overcoming many of the limitations to their performances, further extending their applicability. It would also be interesting to explore this also with other materials, for instance, other transition‐metal (TM) oxides (such as Fe, Co, Mo, Ni, V, Cu, and so on), which exhibit the same potential for large scale application, because they are non‐noble, low cost, and thus promising substitutes of conventional catalysts based on Pt, Pd, Au, Ru, or Ir. In fact, the latter ones are rare, much more expensive, and unlikely to be applied on a large scale or at an industrial level. On the contrary, ZnO, α‐Fe_2_O_3_, and their composites are abundant, cheaper, and easily obtainable on a large scale as well.

## Conflict of Interest

The authors declare no conflict of interest.
